# p53/p73 Protein Network in Colorectal Cancer and Other Human Malignancies

**DOI:** 10.3390/cancers13122885

**Published:** 2021-06-09

**Authors:** Anđela Horvat, Ana Tadijan, Ignacija Vlašić, Neda Slade

**Affiliations:** Laboratory for Protein Dynamics, Division of Molecular Medicine, Ruđer Bošković Institute, Bijenička 54, 10000 Zagreb, Croatia; Andjela.Horvat@irb.hr (A.H.); Ana.Tadijan@irb.hr (A.T.); Ignacija.Vlasic@irb.hr (I.V.)

**Keywords:** p53 isoforms, p73 isoforms, colorectal cancer, p53 family, isoform crosstalk

## Abstract

**Simple Summary:**

The p53 family of proteins comprises p53, p63, and p73, which share high structural and functional similarity. The two distinct promoters of each locus, the alternative splicing, and the alternative translation initiation sites enable the generation of numerous isoforms with different protein-interacting domains and distinct activities. The co-expressed p53/p73 isoforms have significant but distinct roles in carcinogenesis. Their activity is frequently impaired in human tumors including colorectal carcinoma due to dysregulated expression and a dominant-negative effect accomplished by some isoforms and p53 mutants. The interactions between isoforms are particularly important to understand the onset of tumor formation, progression, and therapeutic response. The understanding of the p53/p73 network can contribute to the development of new targeted therapies.

**Abstract:**

The p53 tumor suppressor protein is crucial for cell growth control and the maintenance of genomic stability. Later discovered, p63 and p73 share structural and functional similarity with p53. To understand the p53 pathways more profoundly, all family members should be considered. Each family member possesses two promoters and alternative translation initiation sites, and they undergo alternative splicing, generating multiple isoforms. The resulting isoforms have important roles in carcinogenesis, while their expression is dysregulated in several human tumors including colorectal carcinoma, which makes them potential targets in cancer treatment. Their activities arise, at least in part, from the ability to form tetramers that bind to specific DNA sequences and activate the transcription of target genes. In this review, we summarize the current understanding of the biological activities and regulation of the p53/p73 isoforms, highlighting their role in colorectal tumorigenesis. The analysis of the expression patterns of the p53/p73 isoforms in human cancers provides an important step in the improvement of cancer therapy. Furthermore, the interactions among the p53 family members which could modulate normal functions of the canonical p53 in tumor tissue are described. Lastly, we emphasize the importance of clinical studies to assess the significance of combining the deregulation of different members of the p53 family to define the outcome of the disease.

## 1. Introduction

p53 has a central role in tumorigenesis; it regulates cellular response to stress signals or oncogenic cellular stimuli by inducing transient or permanent cell-cycle arrest, DNA repair, apoptosis, or senescence [1]. The inactivation of the p53 tumor suppressor is the single most common genetic defect in human cancer. The mutations of *TP53* have been found in nearly all tumor types and are estimated to contribute to more than 50% of all cancers.

In the late 1990s, two novel family members *TP73* [2] and *TP63* [3] were described. Both encode several proteins whose structures and functions are similar to those of p53 but not identical, each displaying peculiar functional features revealed by mouse model studies.

**Figure 1:**
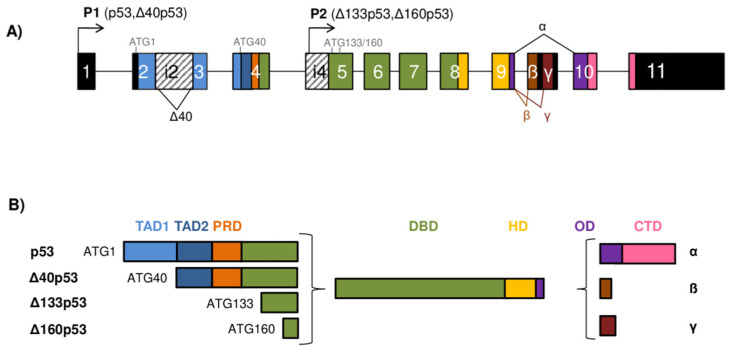
The *TP53* gene architecture and generation of the p53 isoforms. (**A**) The scheme of the *TP53* gene structure. The human *TP53* gene consists of 11 exons and two alternative exons 9β and 9γ. The exons are shown as boxes of different colors and noncoding sequences in black. Introns 2 (i2) and 4 (i4) are shown as boxes with a striped pattern. The *TP53* gene can be transcribed from two different promoters, the canonical promoter P1 upstream of exon 1 (giving rise to the long p53/Δ40p53 isoforms) and the alternative P2 located in intron 4 (giving rise to the short Δ133/Δ160p53 isoforms). The *TP53* mRNAs can be alternatively spliced at intron 2 or intron 9, producing isoforms with different N- and/or C-termini. There are four possible different start codons for mRNA translation (ATG1, ATG40, ATG133, and ATG160) resulting in protein isoforms of varying length. (**B**) Modular structure of the p53 protein isoforms. Functional protein domains are shown in different colors matching those used for the encoding exons of the *TP53* gene in (**A**). The full-length p53α protein consists of two transactivation domains (TAD1 and TAD2), a proline-rich domain (PRD), a DNA-binding domain (DBD), a hinge domain (HD), an oligomerization domain (OD), and a C-terminal domain (CTD). There are 12 distinct p53 protein isoforms differing in the composition of the structural domains. Model adapted from [8,9,10].

**Figure 2:**
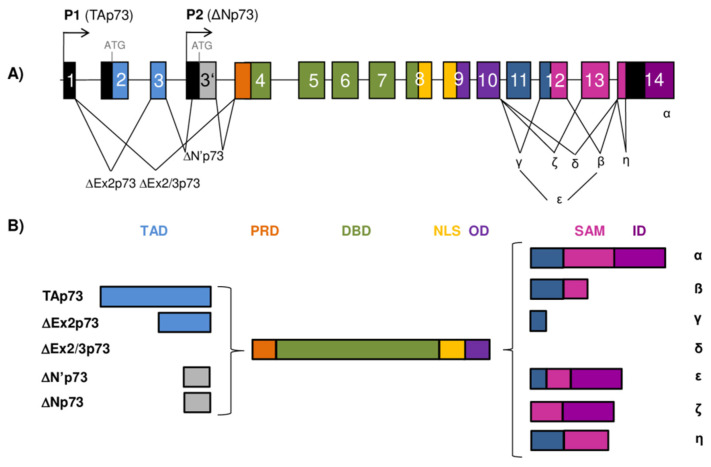
The *TP73* gene architecture and generation of the p73 isoforms. (**A**) The scheme of the human *TP73* gene structure. The human *TP73* gene consists of 14 exons and additional alternative exon 3′ (shown as gray box). The exons are shown as boxes of different colors and noncoding sequences in black. There are two different promoters, the canonical promoter P1 upstream of exon 1 (giving rise to TAp73 isoforms) and the alternative P2 located in intron 3 (giving rise to ΔNp73 isoforms). The P1 transcript can be alternatively spliced leading to the expression of several N-terminally truncated isoforms (ΔEx2p73, ΔEx2/3p73, and ΔN′p73). Alternative splicing is also possible at the C-terminus, giving rise to seven potential isoforms (α, β, γ, δ, ε, ζ, and η). (**B**) Modular structure of the p73 protein isoforms. Functional protein domains are shown in different colors matching those used for the encoding exons of the *TP73* gene in (**A**). The full-length TAp73α protein consists of a transactivation domain (TAD), a proline-rich domain (PRD), a DNA-binding domain (DBD), a nuclear-localization signal (NLS), an oligomerization domain (OD), a sterile α-motif (SAM), and an inhibitory domain (ID). There are 28 possible distinct p73 protein isoforms differing in the composition of the structural domains (as Δ’Np73 and ΔNp73 mRNAs translate into identical proteins).

All members of the p53 family are evolutionarily conserved and have a very high structural similarity within three main functional domains: transactivation domain (TAD), DNA-binding domain (DBD), and oligomerization/tetramerization domain (OD/TD) (Figure 1 and Figure 2). All family members share significant amino-acid homology in DBD (over 60%), which enables them to bind the p53-responsive elements (p53RE) and transactivate a large number of the same target genes. Accordingly, p63, p73, and p53 proteins form a family of transcription factors. At the sequence level, p63 and p73 share higher homology to each other than to p53 [4]. However, despite many overlapping roles, each member has its unique identity and functions.

The family complexity has been enriched by the transcription from alternative promoters, alternative splicing, and diverse translation initiation sites [4,5]. Consequently, several protein isoforms with distinct N- and C- termini are encoded.

The roles of each particular p53 family member were determined by transgenic knockout mice. While the p53 knockout mice show high susceptibility to spontaneous and induced carcinogenesis, thereby defining p53 as an important tumor suppressor, total p63 and p73 knockout mouse models lack a cancer phenotype. Instead, p63- and p73-null mice exhibit various developmental deficiencies revealing their crucial functions in epithelial and central nervous system formation, respectively [4]. Although p63 and p73 are rarely mutated in cancer, *TP63* is situated within the locus frequently amplified in squamous cell carcinoma [6]. Their role in tumorigenesis is defined by several isoforms with opposed functions, in the same cellular context [7]. However, their tumor suppressive potential was not fully elucidated until the establishment of the isoform-specific knockout mice. In this review, we summarize the structural and functional properties of different p53 and p73 isoforms and their roles in tumor formation with the emphasis on colorectal cancer (CRC).

## 2. Gene Architecture and Generation of the p53/p73 Isoforms

The human *TP53* gene is located on the chromosome 17p13.1 and comprises 11 exons and two alternative exons 9β and 9γ. It has a dual gene structure due to the presence of two functional, distinct promoters, the canonical P1 located upstream of exon 1 and the internal P2 that lies within intron 4, from which several *TP53* mRNAs can be transcribed (Figure 1A) [11]. The *TP53* mRNAs can alternatively be spliced at intron 2 or intron 9, resulting in variants with different N- or C-termini. In addition, the *TP53* mRNA can also contain two internal ribosomal entry site (IRES) elements that mediate translation in a cell-cycle phase-dependent manner [12,13]. Translation of the *TP53* mRNA can start at different codons, e.g., codon 1 (ATG1), codon 40 (ATG40), codon 133 (ATG133), or codon 160 (ATG160) [8,14], resulting in p53 isoforms that differ in length (Figure 1B).

Consequently, the p53 isoforms can be classified as long or short depending on the initiation of transcription and translation. Transcribed from the canonical P1, the *TP53* mRNA transcripts translate at codon 1 and/or codon 40 to encode the long isoforms (p53/Δ40p53), while transcribed from the internal P2, translation can start at codons 133 and/or 160, giving rise to the short isoforms (Δ133p53/Δ160p53). The transcription from the canonical P1 generates intron 2-spliced (also known as fully spliced, FSp53) or intron 2-retained (also designated as p53I2) *TP53* mRNA transcripts. Translation at the codon 1 of intron 2-spliced *TP53* mRNA transcript encodes the full-length p53 isoforms [8,14]. However, due to the alternative translation initiation sites and the presence of IRES elements in the 5′ untranslated regions (5′UTR) in the *TP53* mRNA, IRES-mediated translation can produce Δ40p53 isoforms from the codon 40 [12,13,15,16,17]. In addition, Δ40p53 isoforms can also be synthesized from the *TP53* mRNA transcripts with retained intron 2 that are translated only at codon 40 [8,11,14]. Initiation of transcription from the internal P2 within intron 4 generates a *TP53* mRNA transcript that can be translated either at codons 133 or 160, encoding the Δ133p53 or Δ160p53 isoforms, respectively [8,14,18]. The alternative splicing from the exon 9 to either exons 9β or 9γ generates the *TP53* mRNA transcripts that encode β or γ isoforms, respectively. Since both exons 9β and 9γ contain premature termination codons (PTCs), exons 10 and 11 remain untranslated in β and γ isoforms [8,11,14]. To conclude, depending on alternative promoter usage (P1 or P2), alternative splicing of intron 2 or intron 9, and alternative initiation of translation, different p53 isoforms can be generated. Consequently, nine different *TP53* mRNA transcripts encoded by the *TP53* gene can give rise to 12 protein isoforms, known as p53α/β/γ, Δ40p53α/β/γ, Δ133p53α/β/γ, and Δ160p53α/β/γ [8,14]. Essentially, there is an increased expression of the full-length p53 in tumor tissue compared to corresponding normal tissue which is usually attributed to mutations in p53. Depending on the p53 mutation status, the p53 isoforms can be differentially expressed. The increased expression of different N- and/or C-terminal spliced variants has been detected in various cancer entities, such as the Δ133p53α isoform in colon, lung, and ovarian cancer, cholangiocarcinoma, and melanoma [9,14,19,20,21,22] or the Δ40p53α isoform in both glioblastoma and breast cancer [14,23,24]. Furthermore, the p53β isoform is identified and overexpressed in the head and neck squamous cell carcinoma and renal cell carcinoma, respectively [25,26]. Interestingly, the expression profile of the N- and C-terminal spliced variants can differ in premalignant lesion, tumor, and corresponding healthy tissue. For example, premalignant lesions of colon adenomas are shown to express reduced levels of N-terminal splice variant (e.g., Δ133p53α) and elevated levels of C-terminal splice variant (e.g., p53β) compared with normal colon tissues. In contrast, the expression pattern of these splice variants depends on the p53 status and is changed in colon carcinoma tissue compared to colon adenoma, suggesting their role in cancer progression [19,27].

The p73 protein was discovered in 1997 as a product of the *TP73* gene situated in the region 1p36.33 that is often deleted in neuroblastoma and various other human tumors. Frequent occurrence of loss of heterozygosity (LOH) and structural homology with the canonical tumor suppressor p53 placed p73, together with almost concomitantly discovered p63 protein, into the common p53 protein family [2,3]. The human *TP73* gene consists of 15 exons (designated as exons 1–14, plus one alternative exon 3′) and it can be transcribed into different mRNAs, subsequently producing several different protein isoforms (Figure 2). Multiple mRNAs are produced as a result of the existence of two promoters combined with the alternative splicing at the 5’- and 3′-ends. The transcription from P1 located in the 5′UTR upstream of exon 1 gives rise to a group of transcriptionally active TAp73 isoforms. Usage of the alternative P2 situated in the intron 3 produces N-terminally truncated isoforms that are, thus, referred to as ΔNp73 isoforms [28,29,30].

The transcript generated from P1 promoter can be alternatively spliced at the 5’-end, producing more complexity among the N-terminally truncated isoforms. In this way, three additional isoforms are produced, namely, ΔEx2p73 (lacking exon 2), ΔEx2/3p73 (lacking exons 2 and 3), and ΔN′p73 (containing alternative exon 3′) [2,29,30,31]. Interestingly, the ΔN′p73 transcript and the ΔNp73 transcript generated from P2 promoter produce an identical ΔNp73 protein isoform and can be distinguished exclusively on an mRNA level. Specifically, the ΔN′p73 transcript aberrantly contains 198 bp from the alternative exon 3′ leading to a PTC in the regular reading frame, and, as a result, the translation starts at the same codon in exon 3′ used by the P2 transcript [30,32].

Similar to p53, an IRES element was discovered in exon 2 of the *TP73* mRNA representing another possible mechanism of p73 expression regulation via CAP-independent translation [33]. Alternative splicing is even more frequent at the 3′-end, producing seven different transcripts named α, β, γ, δ, ε, ζ, and η [2,29,34,35]. Combining the usage of two different promoters and splicing at 5′/3′-ends can theoretically result in 35 different mRNAs, which can be translated into 28 different protein isoforms, but not all of them have been detected in cell lines or tissues so far. In general, p73 expression is often higher in tumor tissues compared to the corresponding normal tissues. The increase in expression is mostly attributed to the N-terminally truncated isoforms, but the TAp73 isoforms are also found to be increased in several tumors. An increase in the variety of the C-terminally spliced isoforms has been connected to certain tumor types. Thus, normal breast, colon, and myeloid cells predominantly express the p73α and β isoforms, while, in breast and colon cancer, as well as in acute myeloid leukemia, an increased level of the p73γ, δ, and ε isoforms was observed [36,37,38].

## 3. Structure of the p53 Protein Isoforms

The canonical, full-length p53 (p53α) isoform displays a modular domain structure and contains 393 residues that are organized in seven functional domains (Figure 1B). The N-terminal region of the p53 protein contains two distinct acidic transactivation domains (TAD1, residues 1–39 and TAD2, residues 40–61) that are intrinsically disordered, which allows binding with high specificity to their interacting partners [39,40]. Each TAD can transactivate genes independently and are required for the transactivation of different target genes and effector pathways. Through its TAD, the p53 protein interacts with the components of transcriptional machinery (e.g., TBP, TFIIH), proteins involved in DNA metabolism (e.g., PCA, RPA), chromatin modifiers (e.g., p300/CBP, GCN5), and inhibitors of p53 (e.g., MDM2, MDMX). Functional analysis based on in vivo mouse models showed that TAD1 is important for the transactivation of the p53 target genes involved in cellular responses to acute DNA damage, such as apoptosis and cell-cycle arrest, while both TAD1 and TAD2 can cooperate in the transactivation of genes associated with tumor suppression [41,42]. Furthermore, it has recently been shown that the induction of endoplasmic reticulum (ER) stress-inducible genes (e.g., *PTP4A1*, *PLK2*) depends on the transactivation activity of the TAD2 [43].

Following TA domains at the N-terminus lies a proline-rich domain (PRD, residues 62–93) that contains 15 proline residues, some as part of five PXXP motifs (where P represents proline and X any residue) important for growth suppression. These motifs have the ability to bind SH3 (Src homology 3) domains of proteins such as c-Src and PI3K (phosphatidylinositol 3-kinases) and mediate signal-transduction pathways [44,45]. The PRD is shown to control modifications that influence p53 functions, e.g., activity and stability. It contains conformationally flexible motifs that bind transcriptional coactivator p300, involved in p53 activation through acetylation [46]. Furthermore, the PRD is important for regulating p53-mediated apoptosis [47,48]; rs1042522 (p53 p.R72P) is a common polymorphism in the PRD whose allele frequency differs in populations worldwide [49] and was recently associated with the CRC risk among specific ethnic groups according to a meta-analysis [50].

The p53 protein binds to DNA through its core domain, the conserved sequence-specific DBD (residues 94–290). The DBD contains four conserved regions [51] and includes the immunoglobulin-like β-sandwich that serves as a scaffold for the structural elements involved in DNA-binding consisting of two large loops, which are held together by a zinc atom through cysteine and histidine ligands, and a loop–sheet–helix motif [52]. It has been recently shown that the DBD regulates conformation stability of the p53, and its absence, as in the Δ133p53 and Δ160p53 isoforms, can destabilize the structure and trigger aggregation propensity, ultimately causing abnormal protein function [53]. Most cancer-associated mutations occur within the DBD, causing structural alterations (e.g., R175, Y220, G245) or impacting DNA binding (e.g., R248 and R273) [54], which makes DBD crucial for the tumor-suppressive functions of p53. Furthermore, p53-dependent tumor suppression and apoptosis can be modulated by mutations in residues E180 and R181 that provide the structural basis for cooperative binding of p53 to target promoters [55,56].

The p53 protein possesses a nuclear-localization signal (NLS, residues 305–322) that facilitates the shuttling of p53 between the nucleus and cytoplasm. It has been shown that the nuclear import of p53 is negatively regulated by ubiquitination of lysine residues 319–321 within the NLS, causing the retention of p53 in the cytoplasm. Upon stress, the level of ubiquitination reduces, and the positive charges of NLS residues are rendered unmasked and recognized by importin α3, an adaptor molecule that facilitates nuclear import of non-ubiquitylated p53 [57,58]. The NLS is located in the hinge domain (HD, residues 291–324), the short linker between DBD and OD (residues 325–356). Germline mutations in the HD (e.g., p.K305M and p.G325V) have been shown to impair apoptotic functions of mutant (mt) p53 protein that still retains the ability to induce cell-cycle arrest [59]. Additionally, specific missense and deletion mutations in the HD (e.g., p.R306P, p.del300-308 and p.del300-327) cause the loss of apoptotic function and reduced ability of p53 to transactivate the *BAX* promoter, but do not affect the transactivation of *p21* promoter [60], which would imply that the HD is involved in allosteric regulation of DNA binding [61].

To fulfill its function as a tetrameric transcription factor, p53 needs to exhibit its capacity to form tetramers, an active form needed for binding to RE and gene transactivation, dependent on the OD. It has been revealed that the monomer, which consists of a β-strand and an α-helix, connects with the second monomer to form a dimer in an antiparallel manner by means of their β-strands and α-helices. Two dimers connect through their α-helices to form a tetramer. On the other hand, β-strands are not included in the interaction between the dimers since they are located outside of the tetramer [62]. In addition, the OD contains a leucine-rich nuclear export signal (NES, residues 340–351) that regulates subcellular localization of p53 and is masked in the tetramers, permitting their accumulation in the nucleus. Consequently, there seems to be a coordinated regulation of p53 tetramerization and its nuclear retention, which is dependent on the NES placement [63]. It has been recently shown that the positively charged residues within the NES of OD are required for proper regulation of the p53 target genes. For example, changing lysines 351 and 357 to glutamines does not affect localization or tetramerization status of the protein. However, p53 is impaired in the induction of cell-cycle arrest but retains the ability to induce cell death [64]. Many other missense mutations in the OD have been reported [65], some of which can impair the ability of p53 to form tetramers and to activate transcription of its target genes. It has been shown that the p53 OD mutants with the alterations in L330 (i.e., p.L330R, p.L330E or p.L330P) can exhibit severe homotetramerization and ubiquitination defects and the loss of transcriptional activity. However, when co-transfected with wt p53, they could form heterotetramers and alter the expression of p53 target genes (*BAX* and *p21*), thereby acting as dominant-negative mutants [66].

The C-terminal domain (CTD) (residues 357–393) is an arginine- and lysine-rich basic domain that recognizes and binds nonspecifically to DNA and RNA. The CTD is intrinsically disordered and contains NLSs, as well as the sites with the most frequent posttranslational modifications (PTM) that include phosphorylation, acetylation, ubiquitination, methylation, neddylation, and sumoylation [39,40,67,68]. Hence, the CTD regulates the activity of the protein. Initially, the CTD was suggested to be a negative autoregulator of sequence-specific DNA binding. However, later studies have described positive regulatory features of the CTD that likely depend on low-affinity electrostatic interactions between the DNA phosphate backbone and C-terminal lysine residues [40]. It has recently been shown that the CTD is required for the DBD to recognize p53RE, and it is able to modulate structural changes within the DBD, thus stabilizing the association of p53 to DNA-binding sites [40,69].

To summarize, different p53 isoforms, except the canonical full-length p53, lack part of the N- and/or C-termini and, consequently, are deficient in some of the functional domains (Figure 1B). The isoforms with N-terminal deletions have a designation depending on the length of the deletion; hence, considering the lack of the first 39, 132, or 159 residues, they are called Δ40p53, Δ133p53, or Δ160p53 isoforms, respectively. Due to the lack of the first 39 residues, TAD1 is absent in the Δ40p53 isoforms that still retain TAD2. Both TAD and PRD are absent in the Δ133p53 and Δ160p53 isoforms that, to some extent, retain the DBD. The Δ133p53 isoforms lack a small part of the first conserved cysteine box of the DBD, which is completely absent in the Δ160p53 isoforms. However, the Δ160p53 isoforms do retain the other three cysteine boxes of the DBD. The isoforms can exhibit differences in the C-terminus due to alternative splicing of exon 9. The α isoforms contain exons 10 and 11 that encode the OD and CTD. Due to PTCs present in exons 9β and 9γ, both β and γ isoforms lack part of the OD and complete CTD. The first seven residues of the OD are present in all isoforms (α, β, γ); however, the β isoforms contain an additional 10 residues, while γ isoforms contain an additional 15 residues that differ in sequence [8,14].

## 4. Structure of the p73 Protein Isoforms

The full-length p73 and p53 proteins show substantial degree of homology in the composition of the main functional protein domains (TAD, DBD, and OD) (Figure 1B and Figure 2B). The level of homology between p53 and p73 proteins is the highest in their DBDs (63%), emphasizing their central role as transcription factors binding to the promoters of various, many overlapping, target genes. A significant level of homology exists also between their TADs (29%) and ODs (38%) [5]. However, at the C-terminus, there is more diversity between the family members. While p53 contains the CTD at its C-terminus, the p73 protein possesses a unique sterile α motif (SAM) domain, as well as an inhibitory domain (ID). Both p53 and p73 can appear as numerous isoforms with different composition of protein domains, susceptible to different PTMs and regulatory protein interactions. This provides a broad area for investigation in order to offer an explanation for their distinct transcriptional activity and functions [61].

Only the TAp73 isoforms transcribed from P1 that are not subjected to additional splicing at the 5′-end contain a complete TAD at the N-terminus, which makes them strong transcriptional activators (Figure 2B). At first, it was considered that the p73 protein contains only one unique TAD, in contrast to p53 which possesses two [70]. However, more recent work on the interaction between the TAp73 N-terminus and different domains of transcriptional activator p300 revealed the existence of two distinct p73 transactivation subdomains, spanning residues 10–30 and 46–67 [71,72]. In contrast to TAp73, the ΔNp73 isoforms transcribed from P2 in intron 3 lack the first 62 residues and, consequently, do not contain the TAD of the N-terminally intact isoforms. The presence or absence of the TAD, defining these two groups of isoforms, is reflected in their ability to transactivate different genes. While the TAp73 isoforms can activate the expression of various genes, many of which are also induced by p53, the ΔNp73 isoforms are mostly transcriptionally inactive. Moreover, the ΔNp73 isoforms act as dominant-negative inhibitors of p53 and TAp73 [28,31]. However, inducible overexpression of the ΔNp73β isoform was found to induce certain p53/p73 target genes such as *p21*, *14-3-3σ*, and *GADD45* causing cell-cycle arrest, growth suppression, and apoptosis in different cell lines. This transactivation activity was dependent on the presence of 13 unique residues at the N-terminus of the ΔNp73 isoforms and the adjacent region with PXXP motifs (PRD), which together form a novel transactivation domain specific for the ΔNp73 isoforms [73]. Similar activity was also shown for ΔNp73γ, whereas ΔNp73α was inactive in the growth suppression and transactivation [73]. In another study, overexpression of both ΔNp73β and TAp73β induced transcription of the antiapoptotic short caspase-2_S_ isoform, possibly contributing to pro-survival mechanisms in tumors. The induction of caspase-2_S_ expression was dependent on direct TAp73β/ΔNp73β binding to a specific 18 bp site in the *CASP-2S* promoter region. Neither ΔNp73α nor TAp73α was able to induce caspase-2_S_ expression under the same experimental conditions [74]. In contrast, ΔNp73α was able to induce several genes, including *EGR1* (early growth response 1) and *CDC6* (cell division cycle 6), independently of p53 in microarray analysis [75]. In addition, ΔNp73α enhanced the expression of the TGFβ target genes *PAI-1* and *Col1a1*, possibly through its interaction with Smad transcription factors and subsequent binding to Smad-binding elements (SBEs) [76]. In more recent research, ΔNp73β and, to a lesser extent, ΔNp73α were found to enhance the expression of several keratinocyte genes in cooperation with ΔNp63α, having a role in skin development, proliferation, and wound healing [77].

The PRD of the p73 protein comprises two PXXP motifs situated between residues 84–87 and 103–106 [78]. Between the two PXXP motifs, there is Y99, a residue that was shown to be phosphorylated by c-Abl kinase after γ-irradiation, correlating with the induction of p73 target genes and apoptosis [79,80]. In contrast to its importance for ΔNp73β transcriptional activity, neither Y99 nor the PXXP motifs were proven to be indispensable for the activity of TAp73β isoform in the induction of p21 expression and apoptosis [78].

The structure of the p73 DBD is highly similar to that of the p53 protein but differs in the L2 loop important for protein–protein interactions [81]. The DBD of the p73 protein is responsible for its binding to REs of more than 200 different genes involved in important cellular processes such as apoptosis, DNA repair, and neuronal and epidermal differentiation. It was recently shown using a biochemical approach that even small sequence variations in the RE could affect binding affinity and velocity of p73 with its DBD [82].

The OD of p73 comprises residues 352 to 390 and enables tetramerization of p73 and its activity as a transcription factor. In spite of the high degree of similarity in amino-acid sequence with the OD of the p53 protein, the heterotetramers composed of the full-length p73 and p53 have not been identified. This was explained by the discovery of an additional *α*-helix located C-terminally of the core OD (homologous to p53) whose deletion causes a conformational instability and dissociation of the p73 homotetramer into dimers. The absence of the second *α*-helix is reflected in the reduced p73 transcriptional activity [83]. As intact OD is present in all identified p73 isoforms, including both N- and C-terminally truncated, there is a complex network of possible protein interactions to be explored.

Next to the OD, there is a long stretch of 109 amino acids connecting it to an SAM domain which is specific for the C-terminus of p73 [83]. The SAM domain constitutes five helices (α1 to α5) forming a globular structure, and it is considered important for modulation of the p73 transcriptional activity [84]. It is encoded by exons 11, 12, and 13, a region that is subjected to intensive alternative splicing. There have so far been seven different 3′-splice variants discovered (α, β, γ, δ, ε, ζ, and η), which makes p73 the most complex among the p53 protein family members regarding alternative splicing at the 3′-terminus [85]. The C-terminally truncated p73 isoforms are produced as a consequence of the PTCs created by alternative splicing. Only α isoforms of p73 contain a complete SAM domain. As a consequence of alternative splicing, the p73β isoforms lack exon 13. Although the p73γ isoforms contain all the exons coding for the SAM domain, the usage of the long alternative frame due to splicing at exon 11 leads to a PTC. The p73δ isoforms lack most of the p73-specific C-terminal region (exons 11–13), resembling p53 more than other p73 isoforms [34]. The p73ε isoforms contain parts of the p73γ and p73α reading frames lacking exons 11 and 13. The p73ζ isoforms are produced by internal deletion of exons 11 and 12, lacking functionally important parts of the SAM domain [35]. The p73η isoforms are closely related to p73α but differ at exon 14. Next to the SAM domain, at the very C-terminus, the p73α, p73ε, and p73ζ isoforms have an additional ID. The differences in the presence and functionality of the SAM and ID have been shown to modulate the transcriptional activity of the C-terminally spliced p73 isoforms. In that sense, the TAp73β isoform, which lacks both the SAM domain and the ID, is a stronger transcriptional activator than TAp73α, which contains both. Although, in TAp73α, the ID does not directly interact with the TAD, it prevents association with the transcriptional coactivator p300/CBP, contributing to inhibition of its transcriptional activity [86].

The differential structure of multiple p53/p73 isoforms and their potential interactions offer a broad area for investigation of the molecular basis for their diverse biological functions.

## 5. Regulation of the p53 Isoforms’ Expression and Activity

**Figure 3:**
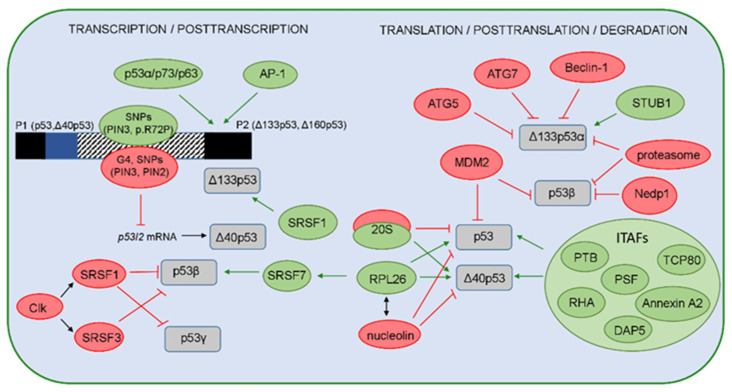
A model representing regulation of the p53 isoforms’ expression and stability. The positive regulators are shown in green, while the negative regulators are shown in red. On the transcriptional level, expression of the p53 isoforms is regulated by usage of two different promoters P1 or P2, producing the long (p53, Δ40p53) or the short (Δ133p53, Δ160p53) isoforms, respectively. Several regulators can influence the P2 activity. The canonical p53 and p53 family members (p63 and p73 and their isoforms) are shown to transactivate P2 with different efficiency. The transcription from P2 can be activated by the AP-1 transcription factor that mediates the expression of Δ133p53 in *H. pylori*-infected cells. The single-nucleotide polymorphisms (SNP) and their haplotypes in the internal promoter region (shown as box with a striped pattern) that comprises intron 3, exon 4, and intron 4 can affect the P2 activities. Furthermore, the G4 structures, PIN3 and PIN2 polymorphisms, can decrease the level of the p53I2 mRNA that encodes the Δ40p53 isoforms. The p53 isoforms are regulated on the posttranscriptional level by different splicing factors. SRSF1 and SRSF3, activated by Clk, promote complete exclusion of intron 9 and, thus, negatively regulate the level of p53β and p53γ isoforms. However, SRSF1 upregulates the Δ133p53α expression in human aortic smooth muscle cells. In addition, the binding of RPL26 to the *TP53* pre-mRNA allows the recruitment of SRSF7 that prompts alternative splicing and, thus, generates p53β isoforms. Due to IRES, the level of p53 and Δ40p53 is regulated by ITAFs (PTB, Annexin A2, PSF, DAP5, TCP80, RHA) or proteins such as RPL26 or nucleolin. Interestingly, Δ40p53 can be generated by the 20S proteasome that degrades the full-length p53 protein. The level of the full-length p53 protein is regulated by MDM2 that was shown to promote the degradation of p53β. In addition, the level of p53β is also regulated by the MDM2-dependent neddylation, proteasome, and deneddylating enzyme Nedp1. The level of the Δ133p53α isoform is regulated by the proteasome, as well as via autophagic degradation, upon replicative senescence, where the proautophagic proteins (ATG5, ATG7, Beclin-1) act as positive regulators, while the STUB1/CHIP acts as a negative regulator of Δ133p53α degradation and senescence.

The expression and function of the p53 isoforms can be regulated on the transcriptional, posttranscriptional, translational, and posttranslational levels (Figure 3).

As previously mentioned, the expression of p53 isoforms on the transcriptional and posttranscriptional levels is regulated by *TP53* promoter usage (P1 or P2) and the alternative splicing of intron 2 or 9, which can be modulated by different factors. The expression of p53 isoforms is tissue-specific and can be precisely regulated. In addition to epigenetic events that influence the activity of *TP53* promoters [11], several single-nucleotide polymorphisms (SNP), more specifically, their haplotypes, can modify the activity of the internal P2 promoter, thereby affecting the expression of p53 isoforms. There are eight SNPs, including common p53 p.R72P and PIN3 Ins16bp (16 bp insertion in intron 3, rs17878362), in 11 different haplotypes identified within the P2. Using specific reporter gene assay constructs, two of the 11 haplotypes were shown to increase the baseline expression of Δ133p53 isoform [87]. In addition, a heterozygous combination of SNPs in the P2 promotor region (such as a combination of p53 p.R72P with either SNP in intron 4, i.e., rs9895829 or rs2939430) can affect the expression of Δ133p53, as well as the p53β isoform [88]. Furthermore, it has been previously shown that different p53 family members and their isoforms can regulate the expression and function of Δ133p53 isoforms [89,90,91,92]. The transcription from P2 can specifically be activated by the AP-1 (activator protein-1), a c-Jun/c-Fos transcription factor, which upregulates the expression of the Δ133p53 isoform in *Helicobacter pylori*-infected gastric epithelial cells [93].

On the posttranscriptional level, several factors have been shown to regulate the alternative splicing of intron 2 or 9. The splicing factors, such as SRSF1 and SRSF3, which are members of serine/arginine-rich (SR) proteins, have been shown to negatively regulate the expression of p53β and p53γ isoforms [91,94]. SR proteins are essential for spliceosome assembly and are activated by Clk (Cdc2-like kinases) [95,96]. In accordance, treatment with Clk inhibitor TG003 and silencing of SRSF1 promote the generation of both *TP53* mRNA β and γ variants and decrease the level of *TP53* mRNA α variants, which would suggest that SRSF1 regulates alternative splicing of *TP53* intron 9 and favors its complete exclusion (e.g., exons 9β and 9γ) [91]. Furthermore, downregulation of SRSF3 increased the level of *TP53* mRNA β variants and induced replicative senescence in early passages of normal human fibroblasts, thus revealing that SRSF3 regulates alternative splicing of *TP53* intron 9 and modulates the p53-mediated cellular senescence [94]. Alternative splicing and the generation of p53β can be regulated by the DNA damage response (DDR) pathway induced after external DNA damage (e.g., ionizing radiation (IR) and alkylating agent MMS). IR was shown to suppress the kinase activity of the hSMG-1 protein, a member of the PI3K family involved in the nonsense-mediated mRNA decay (NMD) pathway, promoting the binding of RPL26 (ribosomal protein L26) to *TP53* pre-mRNA. This allows the recruitment of the splicing factor SRSF7 that prompts the alternative splicing of *TP53* pre-mRNA to generate the p53β isoform, shown to be involved in the regulation of IR-induced cellular senescence [97,98]. In addition to their role in regulating alternative splicing, splicing factors can also regulate the transcription of p53 isoforms. Indeed, using human aortic smooth muscle cells, it has been recently shown that SRSF1 upregulates Δ133p53α expression, but does not alter the expression of full-length p53 or the Δ40p53 isoform [99]. The Δ40p53 isoforms can be generated by alternative splicing of intron 2, which can be affected by the G-quadruplex (G4) structures located in the GC-rich region of intron 3 in *TP53* pre-mRNA [100]. The G4 structures are formed by stacking G-quartets on top of each other, where each of G-quartet contains four guanine bases linked via Hoogsteen hydrogen bonds stabilized by a specific cation, such as a potassium K^+^ ion. The G4 structures can arise in both DNA and RNA sequences and, thus, can affect gene transcription, mRNA splicing, and translation. Indeed, it has been shown that G4 structures promote the splicing of intron 2, resulting in fully spliced *TP53* mRNA. In contrast, mutations that abolished G4 formation prefer retention of the intron 2 and, consequently, increase the expression of intron 2-retained *TP53* mRNA. Therefore, the G4 structures influence the splicing of intron 2 and regulate the ratio between intron 2-spliced and intron 2-retained mRNAs [100]. In addition, the common polymorphism PIN3 was shown to overlap with sequences included in G4 formation, and it can form a quasi-identical G4 structure as the wt allele. PIN3 modulates the level of full-length p53 and Δ40p53 depending on the cell context and was shown to be associated with increased cancer risk depending on the population and cancer entity [101,102,103]. Furthermore, the presence of a polymorphism in intron 2 (rs1642785, PIN2) reduced the stability of p53I2 mRNA and *TP53* pre-mRNA [101]. It has been recently shown that the presence of DNA sequences prone to G4 structures adjacent to p53RE impact the transcriptional activity of p53 family members and their α isoforms (p53/Δ40p53/p73/ΔNp73/p63/ΔNp63); thus, G4 could be an important transcriptional regulatory element [104,105].

On the translational level, p53 isoforms, such as full-length p53 and Δ40p53, can be regulated by IRES-mediated translation [12,17,106], which is increased under different stress conditions that induce DNA damage (e.g., IR, etoposide, doxorubicin) [106,107,108], serum starvation, ER stress [17], glucose deprivation [109], or oncogene-induced senescence [110]. IRES-mediated translation is regulated by ITAFs (IRES-interacting trans-acting factors) such as PTB (polypyrimidine tract-binding protein), Annexin A2, PSF (PTB-associated splicing factor), DAP5 (death-associated protein 5), TCP80 (translational control protein 80), and RHA (RNA helicase A) that drive translation of the full-length p53 and Δ40p53 [108,111,112,113,114]. Interestingly, the SNPs that occur naturally in 5′UTR in *TP53* can cause reduced binding of PTB to the IRES element and weaker IRES activity [115]. Furthermore, *TP53* translation is controlled by several other proteins such as RPL26 or nucleolin, which interact with each other and utilize 5′ or 3′UTRs of *TP53* mRNA to enhance or suppress the p53 translation after stress, respectively [107,116]. In addition to alternative translation, Δ40p53 generation has recently been shown to also be regulated on the posttranslational level. A novel cellular mechanism has been described and includes the activity of the 20S proteasome, whose function is not restricted to complete degradation of proteins but instead involves cleaving some proteins at specific sites, thereby forming functional cleavage products. Indeed, the 20S proteasome can cleave the p53 protein precisely at position 40, generating the Δ40p53 isoform [117,118] that is capable of forming functional heterotetramers with p53 and TAp73, consequently modulating the transcriptional activity of p53/p73 and attenuating the expression of the p53 target genes [117,119,120]. Furthermore, under oxidative stress conditions, enhanced p53 degradation by the 20S proteasome results in the increased level of Δ40p53 isoform [117,118].

Many different PTMs, such as phosphorylation, acetylation, ubiquitination, methylation, neddylation, and sumoylation, can occur at more than 50 sites located within the TAD, DBD, OD, and CTD of the p53 protein where the TAD and CTD are known to be the most affected [67,68]. The CTD contains carboxy-terminal lysines that are targets for MDM2-mediated ubiquitination. MDM2 binds p53 at the N-terminus (i.e., residues 17–23 in the TAD1 [121], promotes ubiquitin-dependent proteasomal degradation, and is critical for maintaining the p53 level, thereby regulating the stability and transcriptional activity of the full-length p53 protein (p53α isoform) [67,122]. However, p53 isoforms are shown to be differentially modified by MDM2 [123]. Although MDM2 can form a protein complex with other p53 isoforms in addition to p53α, such as p53β and p53γ, it promotes ubiquitination and degradation of p53β only. However, MDM2-promoted degradation of p53β seems to be independent of ubiquitination. In addition, ubiquitination and degradation of the p53 isoforms, such as p53β/γ and Δ133p53α/β/γ, can proceed in an MDM2-independent manner, and these processes are in part regulated by the proteasome. Furthermore, MDM2 protects p53β from degradation by the proteasome through promoting neddylation, a process that is negatively regulated by the deneddylating enzyme Nedp1 [123]. The level of Δ133p53α isoform can be regulated through autophagic degradation upon replicative senescence. Both pharmacological inhibition of autophagy by bafilomycin A1 and silencing of proautophagic proteins, such as ATG5, ATG7 and Beclin-1, have been shown to restore Δ133p53α expression. Furthermore, the level of Δ133p53α expression is regulated by the chaperone-associated E3 ubiquitin ligase STUB1/CHIP, which was shown to protect the Δ133p53α from autophagic degradation and, thus, acts as a negative regulator of autophagy and replicative senescence. Interestingly, STUB1 was reported to interact with Δ133p53α and take part in its ubiquitination [124]. Although both Δ133p53 and Δ160p53 isoforms are encoded by the Δ133p53 mRNA transcript [18], recent findings have shown that the Δ160p53 isoform can also be translated from mutated full-length *TP53* mRNA; however, the underlying mechanism still needs to be elucidated [125].

To conclude, we present some regulators and mechanisms that, on different levels, can modulate the expression and stability of p53 isoforms, thereby influencing their biological activities and functions.

## 6. Regulation of the p73 Isoforms’ Expression and Activity

**Figure 4:**
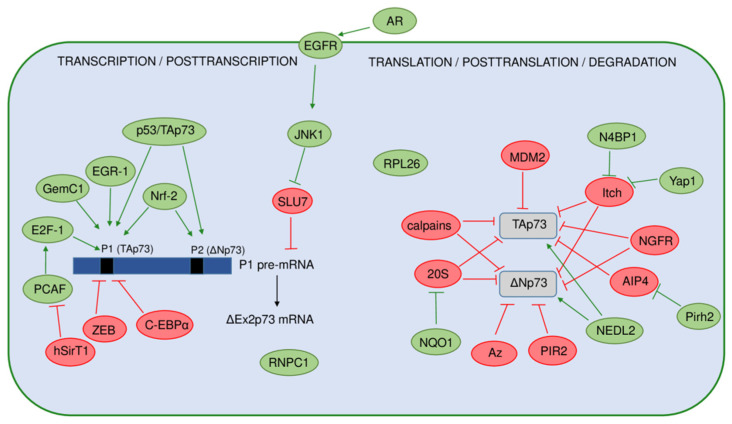
A model representing regulation of the p73 isoforms’ expression and stability. The positive regulators are shown in green, and the negative regulators are shown in red. On the transcriptional level, expression of the p73 isoforms is regulated by usage of two different promoters (P1 and P2) producing the TAp73 or ΔNp73 isoforms. Black bars represent the *TP73* gene promoters. Several regulators specifically activate the P1 promoter, with transcription factor E2F-1 being the most important. In contrast, transcription from P1 is repressed by ZEB, C-EBPα, and hSirT1. The full-length p53 and the TAp73 proteins, as well as Nrf-2, have been found to induce both promoters. On the posttranscriptional level, the alternative splicing of P1 pre-mRNA, leading to increased expression of the ΔEx2p73 isoform, is induced by the activation of EGFR by its ligand amphiregulin (AR) in hepatocellular carcinoma cells. The expression of the ΔEx2p73 isoform is enabled by the inhibition of the RNA splicing factor SLU7 through JNK1 signaling. The stability of the p73 mRNA is increased by the RNA-binding protein RNPC1. Ribosomal protein RPL26 has been found to regulate the p73 translation and protein stability. The p73 protein isoforms are also extensively regulated on a posttranslational level. Here are shown different regulators of the TAp73 and ΔNp73 isoforms’ stability and degradation. Some of them target both TAp73 and ΔNp73 isoforms including Itch, NGFR, calpains, and NQO1 (through 20S proteasome). On the contrary, antizyme (Az) pathway and ligase PIR2 specifically target the ΔNp73 isoforms for degradation after DNA damage.

The p73 expression and activity are regulated at multiple levels, including the previously described usage of multiple promoters, alternative splicing, and translation initiation sites, involving epigenetic and PTMs, as well as interactions with other proteins (Figure 4).

The p73 expression is intensively regulated transcriptionally in response to different stimuli such as DNA damage, viral oncogenes, proliferative signals, and epigenetic modifications [126,127,128,129,130,131]. The main transcriptional factor inducing the expression of the *TP73* gene is E2F-1, which affects only P1-driven transcription. This regulation is crucial for the induction of apoptosis in the absence of functional p53 and is not dependent on posttranscriptional regulation by p14ARF and MDM2 [132,133]. Histone acetyltransferase PCAF (P300/CBP-associated factor) stabilizes E2F-1 by acetylation, leading to an increase in TAp73 expression [127]. The same group later discovered that the regulation of p73 expression by E2F-1 could be modulated by NAD^+^-dependent histone deacetylase hSirT1 through its interaction with PCAF. The interaction of hSirT1 and PCAF represses TAp73 expression as they are co-recruited with E2F-1 on the P1 promoter [134]. The E2F-1-mediated p73 transactivation is also inhibited by the transcriptional repressors C-EBPα and ZEB, both affecting P1 [130,135]. More precisely, a 1 kb regulatory fragment was identified within intron 1 of the *TP73* gene just upstream of the initiating codon ATG in exon 2, which contains six consensus ZEB-binding sites [130]. Later discovered, a polymorphic deletion of 73 bp in this region was detected in normal and tumor tissue, with a higher frequency in patients with breast and colorectal cancer compared to healthy controls. The presence of the allelic variant correlated with higher *TP73* expression in the tumors [136]. Moreover, the same group later reported association of the absence of the 73 bp fragment with adverse clinicopathological parameters in a cohort of colorectal cancer patients [137]. In addition to E2F-1, the EGR1 transcription factor, which is quickly induced as a response to various environmental conditions, also activated the transcription of the TAp73 isoforms [138]. More recently, Nrf-2 (nuclear factor erythroid 2-related factor 2) was discovered to bind both P1 and P2 inducing the transcription of TAp73 and ΔNp73 isoforms. Both Nrf-2 putative binding sites contain CpG methylation islands; however, they showed opposite behavior toward demethylation in breast cancer [139]. GemC1 (geminin coiled-coil domain-containing protein 1) was recently recognized as the transcriptional activator of TAp73 in different multiciliated epithelia. GemC1 interacts with both the E2F-5 transcription factor and the p73 protein, forming a trimeric complex important for the induction of *TP73* promoter activity [140].

Although the p73 transcripts are subjected to intensive 5′- and 3′-terminal splicing, the mechanisms governing the generation of particular splice variants are still poorly understood. It was found that the expression of ΔEx2p73 isoform is induced by activation of EGFR (epidermal growth factor receptor) by its ligand amphiregulin in human hepatocellular carcinoma cells and normal hepatocytes. Generation of the ΔEx2p73 isoform is dependent on the activation of JNK1 (c-Jun N-terminal kinase 1) and the downregulation of the mRNA splicing factor SLU7 [141]. Regarding the regulation of p73 mRNA stability, the RNA-binding protein RNPC1 recognizes and binds to the CU-rich element in the 3′UTR of p73, contributing to the stability of p73 mRNA and increasing its expression. RNPC1 itself is a target of p53 and p73, thus forming a novel feedforward loop [142]. Recently, it was discovered that the ribosomal protein RPL26, which was previously shown to regulate p53 translation, can control p73 mRNA translation and protein stability [143].

The p73 protein is subjected to numerous PTMs (mainly phosphorylations and acetylations) throughout its functional domains, which modulate its activity and stability [144,145,146]. Steady-state levels of the p73 protein are kept low under normal physiological conditions by different mechanisms mainly involving the ubiquitin-proteasome system. Several ubiquitin E3 ligases have been implicated in the regulation of p73 protein stability and degradation [147]. Although MDM2 ligase, the main regulator of the p53 protein turnover, was found to bind and inactivate the p73 protein, it does not cause its proteasomal degradation [148,149,150,151]. The first ligase identified causing the p73 protein degradation was Hect ubiquitin-protein ligase Itch which binds and ubiquitinates both TAp73 and ΔNp73 isoforms, mediating their proteasomal degradation [152]. In contrast, N4BP1 (Nedd4-binding partner-1) and Yap1 (Yes-associated protein 1) abrogate p73 degradation by Itch through their interaction with Itch and p73 itself, respectively [153,154]. Another member of the Itch family, Nedd4 ligase, was found to bind both TAp73 and ΔNp73 isoforms, but was unable to cause their degradation due to its inability to catalyze their ubiquitination. On the other hand, another ligase, NEDL2, was able to bind and ubiquitinate both isoforms; however, instead of degradation, it caused their stabilization [152]. Itch protein level is decreased after DNA damage, causing the stabilization of TAp73 but not ΔNp73. As ΔNp73 was degraded after DNA damage independently of Itch, it implicated another mechanism of its regulation. Later discovered, the ring finger ubiquitin ligase PIR2 was able to differentially control the TAp73 and ΔNp73 protein stability [155]. While the PIR2 expression itself is strongly induced by TAp73, PIR2 causes selective degradation of ΔNp73 after DNA damage, increasing the TAp73/ΔNp73 ratio, which is indicative of apoptosis induction and chemosensitivity of tumor cells in general. PIR2 forms a strong interaction with ΔNp73, inducing its ubiquitination and subsequent proteasomal degradation [155]. Another mechanism of selective ΔNp73 degradation after DNA damage is c-Jun-dependent and mediated by the antizyme pathway [156]. The newest findings reveal the complex regulation of the p73 function and degradation by the interplay of two different E3 ligases, namely, Pirh2 and AIP4. Pirh2 is recognized as a key regulator of AIP4 that was previously found to inhibit p73 function by promoting its ubiquitination and degradation [157]. The level of p73α isoforms that contain the SAM domain can be regulated by NQO1 (NAD(P)H quinone oxidoreductase 1) through a ubiquitination-independent mechanism mediated by 20S proteasome. This pathway is also involved in p53 regulation. NQO1 physically interacts with p73α, p53, and the 20S proteasome, impeding p53/p73α 20S proteasomal degradation [158]. In addition to the proteasome-mediated degradation, the levels of different p73 isoforms can be regulated by calpains which are found to cleave p73 at two possible sites (located in the TAD and OD) [159]. The calpain-dependent regulation of p73 protein level is implicated in the chemosensitivity of ovarian cancer cells after cisplatin treatment through modulation of apoptosis [160]. Recently, a novel mechanism of p73 degradation was discovered through the NGFR (nerve growth factor receptor)-facilitated chaperone-mediated autophagy. NGFR directly binds the DBD of p73 and suppresses its transcriptional activity [161].

Numerous regulators and mechanisms of the p73 isoforms’ expression and stability have been discovered to have an impact on their activity and biological functions. Of particular interest are the mechanisms involved in differential isoform regulation, especially those affecting the TAp73/ΔNp73 ratio, which could be used as a potential biomarker and basis for targeted therapy.

## 7. Biological Activity and Functions of the p53 Isoforms

p53 is a transcription factor, which, via multiple mechanisms, regulates the transcription of a plethora of target genes whose products exert various biological functions [162,163]. The diverse biological activities and functions of p53 can be explained in part by the expression of p53 isoforms and their potential interactions in a tissue-specific manner [11]. Based on the results of numerous studies, including clinical studies, in vivo models, and in vitro cellular models, it seems that the cellular response of p53 corresponds to the co-expression of p53 isoforms and their interactions, thus influencing different pathways. Consequently, the unbalanced expression of p53 isoforms can cause cancer, premature aging, inflammation, developmental disorders, or deficiency in tissue regeneration [8,14].

To investigate biological activities of p53 isoforms, several small interfering RNAs have been designed that specifically target a subgroup of p53 splice variants. Furthermore, different antibodies that recognize the epitopes within different functional domains of p53 have been developed which can detect specific subgroups of p53 protein isoforms [8].

Due to the highly conserved structure of p53 through evolution, different in vivo models (zebrafish, *Drosophila*, mouse) have been developed to investigate the biological activities of p53 isoforms and their involvement in different cellular processes that are associated with tumorigenesis [10,14,164,165]. To date, many biological functions involving p53 isoforms have been described and, depending on the cell/tissue and origin/type (e.g., malignant/nonmalignant, human/mouse/zebrafish, skin/colon/lung/prostate/breast/brain, etc.), they include cell-cycle regulation [11,19,90,99,120,166,167,168,169,170], apoptosis [11,91,120,170,171,172,173,174,175,176], senescence [19,27,165,166,167,177,178], DNA repair [167,176,178,179,180], pluripotent and embryonic stem-cell regulation and cancer stemness [171,181,182,183], normal and cancer cell metabolism [109,184,185], autophagy [186], proliferation [99,175,187,188,189,190], cellular invasion, migration, and angiogenesis [188,190,191,192], immunosuppression [193,194], and inflammation [189].

It has been shown that p53 isoforms can regulate cell fate outcome in response to stress signals by differentially modulating target gene expression in a p53-dependent and p53-independent manner. For example, the zebrafish *TP53* gene (*Zp53*), in addition to Zp53α, encodes Zp53β, ZΔNp53α, and ZΔ113p53α, homologous to human p53β, Δ40p53α, and Δ133p53α, respectively. In response to stress signals, ZΔ113p53α is transactivated by the full-length Zp53 and interferes with Zp53 functions by modulating the expression of p53 target genes, thereby antagonizing apoptotic activity via induction of antiapoptotic *bcl2L* expression and by interacting with p53 [173,174]. Comparable activity is observed in human cells, where Δ133p53 was shown to act as a negative modulator of p53 activity. It has been shown that p53-mediated apoptosis is reduced in cells co-transfected with Δ133p53 and p53 [11]. In addition, upon low doses of doxorubicin, full-length p53 induces Δ133p53 expression which antagonizes p53-dependent apoptosis and G1 cell-cycle arrest, without altering p53-dependent G2 cell-cycle arrest. These effects could be explained by the ability of Δ133p53α to directly interact with p53α and to differentially modulate the expression of p53 target genes *p21*, *MDM2*, and *Bcl-2* [90]. In addition, when overexpressed, Δ133p53 displaces p53α from promoters of p53-inducible genes, such as *p21*, *miR-34a*, *BAX*, and *PUMA* [181]. Interestingly, ZΔ113p53/Δ133p53 were shown to bind novel p53RE in the promoters of DNA repair genes (e.g., *rad51/RAD51*, *lig4/LIG4*, and *rad52/RAD52*) [179,180]. Comparable to Δ133p53, Δ40p53 was also shown to act in a dominant-negative manner toward p53α and, when co-transfected with p53, can impair p53-mediated transcriptional activity, apoptosis, and growth suppression [16,195,196]. Furthermore, in response to ER stress, Δ40p53 and p53α were shown to have different effects on the cell cycle. While p53α induces G1 cell-cycle arrest, Δ40p53 was shown to induce G2 cell-cycle arrest via upregulation of 14-3-3σ [197]. However, high expression of Δ40p53 in Δ40p53-lentivirus-infected melanoma cells was shown to activate endogenous p53 and form Δ40p53/p53 heterotetramers that alter promoter occupancy of apoptotic gene *PIDD* and cell-cycle arrest gene *p21*, thereby promoting apoptosis over cell-cycle arrest upon γ-irradiation [172]. The p53β isoform was shown to differentially bind to promoters, and it can enhance expression of p53 target genes in a promoter-dependent manner. For example, while endogenous p53α preferentially binds to *p21* or *MDM2* promoters rather than the *BAX* promoter, endogenous p53β preferentially binds to *BAX* or *p21* promoters rather than the *MDM2* promoter. In addition, when co-expressed with p53, p53β enhances p53 transcription activity on *BAX*, but not *p21*. Contrary to Δ133p53α and Δ40p53, p53β enhances p53-mediated apoptosis [11].

Increasing evidence implicates Δ133p53α and p53β as regulators of cellular senescence [19,27,165,166,167,177,178]. The characteristics of cellular senescence include elevated expression of cell-cycle arrest markers (*p21*, *miR-34a*, *p16^INK4a^*), increased senescence-associated β-galactosidase activity, and a senescence-associated secretory phenotype (SASP) that includes secretion of proinflammatory SASP cytokines such as IL-6 and IL-1β. Several studies have reported the specific senescent-associated signature of p53 isoform expression that is characterized by reduced expression of Δ133p53 and elevated expression of p53β [19,166,167,177,178]. The senescent-associated signature of p53 isoform expression was observed in different human cells that underwent senescence, such as normal human fibroblasts, T-lymphocytes, astrocytes, and primary fibroblasts derived from Hutchinson–Gilford progeria syndrome (HGPS) patients [19,166,167,177,178]. Interestingly, cellular senescence can be induced by Δ133p53 knockdown and/or overexpression of p53β [19,166,177]. However, the reconstituted expression of Δ133p53 was shown to extend replicative lifespan and to delay senescence [166,167,177,178]. Interestingly, diseases (colon adenoma and neurodegenerative diseases, such as Alzheimer’s disease and amyotrophic lateral sclerosis) with senescence features were also shown to have a senescent-associated signature of p53 isoform expression [19,177].

Upon DNA damage, full-length p53 regulates the expression of its target genes involved in cell-cycle arrest, DNA damage repair, apoptosis, or senescence to maintain genome stability. However, p53α promotes only some DDR pathways, such as excision repair pathways (BER, MMR, NER), while inhibiting double-strand break (DSB) repair mechanisms, such as homologous recombination (HR), nonhomologous end joining (NHEJ), and single-strand annealing (SSA) [198,199,200,201,202,203]. Furthermore, p53 was shown to repress *RAD51* expression and inhibit RAD51 foci formation in response to DNA damage [204]. In addition, p53 represses E2F-1 [205], a positive regulator of RAD51 expression under DNA damage conditions [206]. Interestingly, both ZΔ113p53 and Δ133p53 are strongly induced upon DNA damage and are shown to promote DSB repair via upregulating the expression of key DSB repair genes, such as *rad51/RAD51* (needed for HR), *lig4/LIG4* (needed for NHEJ) and *rad52/RAD52* (needed for SSA) [179]. Although such transcriptional regulation seems to proceed independently of full-length p53, the Δ133p53-mediated DSB repair is coordinated with p73 [180]. It has been shown that Δ133p53 can form a complex with TAp73 [119,180] and, when co-expressed Δ133p53 and p73, can act synergistically to promote the expression of key DSB repair genes, thereby significantly promoting all three DSB repair mechanisms [180]. Furthermore, depending on the cell context, Δ133p53 was shown to facilitate DSB repair by increasing the level of RAD51, a component of HR, via upregulation of E2F-1 in HGPS fibroblasts [167] or by enhancing the repair of 53BP1-positive foci, a component of NHEJ, in irradiated astrocytes [178].

The p53 isoforms are also involved in induced pluripotent stem cell (iPSC) and embryonic stem cell (ESC) regulation. It has been previously reported that overexpression of p44 (homologous to human Δ40p53) impairs the regenerative capacity of adult stem cells while enhancing the regenerative capacity of ESC in mouse models [171,183]. Specifically, Δ40p53 was shown to regulate the level of the IGF-1 receptor, thereby controlling the switch from pluripotent ESCs to differentiated somatic cells by regulating IGF signaling [171]. Interestingly, human iPSCs and ESCs show elevated levels of Δ133p53 compared to p53α and reduced levels of p53-inducible senescence-mediating genes (e.g., *p21*, *miR-34a*, *PAI-1*, *IGFBP7*) [181]. In line with this, the overexpression of Δ133p53 in human fibroblasts repressed the expression of p53-inducible senescence-mediating genes and enhanced reprogramming efficiency to iPSCs that are characterized by lower rates of somatic mutations and reduced chromosomal aberrations compared to iPSCs derived from p53 knockdown fibroblasts [176,181]. Since the tumorigenicity of iPSC represents a serious safety concern, iPSCs derived from overexpressed Δ133p53 have better genetic quality [176,181] and could have a strong potential in regenerative medicine.

Recent research has shown that the p53 family of proteins is involved in the transactivation of metabolic enzymes included in glucose, nucleotide, amino-acid, and lipid metabolism, as well as mitochondrial metabolism and autophagy [207,208,209]. Consequently, in human tumors, particularly CRC, the loss of tumor suppressor activity of p53 and changes in signaling pathways associated with oncogenes *MYC*, *HIF*, and *KRAS*, as well as PI3K/AKT/mTOR axis, are known to play a role in deregulated cellular energetics, an established hallmark of cancer [210,211,212]. Interestingly, evidence points to the involvement of particular p53 isoforms in normal and cancer cell metabolism. For example, it was shown that Δ133p53 binds and utilizes the TAD of ΔNp63 to upregulate the expression of GLUT1 and 4 (glucose transporters), as well as the expression of PGM1 (phosphoglucomutase-1), which drives glycolysis in tumor cells [184]. It has been shown that the oxidized form of vitamin C is structurally similar to glucose and is efficiently taken up via GLUT1 transporters by highly glycolytic cancer cells, such as *BRAF* or *KRAS* mutant CRC cells. Once inside the cell, vitamin C elevates reactive oxygen species (ROS) levels that inactivate glycolytic enzyme GAPDH (glyceraldehyde 3-phosphate dehydrogenase), ultimately leading to an “energy crisis” and cancer cell death [213,214]. Accordingly, due to overexpressed GLUT1 transporters, tumors overexpressing both Δ133p53 and ΔNp63 show increased uptake and higher sensitivity to vitamin C, a feature potentially exploitable for cancer treatment [184]. The Δ40p53 isoforms have also been shown to be implicated in glucose homeostasis of normal and cancer cells. Indeed, loss of Δ40p53 reduced β-cell proliferation and impaired glucose homeostasis in mice leading to diabetes and premature death [185].

Many biological activities of the p53 isoforms have been described in the context of cancer biology. Here, we present only some of the studies that investigated such cellular processes. One is angiogenesis that is inhibited by full-length p53. Bernard and coworkers investigated the role of Δ133p53 in tumor angiogenesis and tumor progression using human glioblastoma cell line U87. The results showed that Δ133p53α and Δ133p53γ but not Δ133p53β promote angiogenesis, while the ratio between p53 and Δ133p53α is important for its regulation. Furthermore, depletion of Δ133p53 isoforms inhibited angiogenesis and glioblastoma tumor growth in in vivo models (chicken chorioallantoic membrane and mice xenografts) [190]. The role of Δ133p53 in promoting invasion and metastasis was confirmed using a mouse model. The Δ122p53 (homologous to human Δ133p53)-expressing cells increased the migratory phenotype that was shown to depend on SASP factors (proinflammatory cytokine IL-6 and chemokine CCL2). Furthermore, Δ122p53 promoted lung metastasis in a B16 mouse melanoma metastatic model [191]. Recent findings correlated Δ133p53β expression with an immunosuppressive environment and chemoresistance. For example, prostate cancers with increased Δ133p53β were shown to exhibit an immunosuppressive phenotype (e.g., increased PD-1, PD-L1, CSF1R cells) and immune cell infiltration (such as T cells and CD163^+^ macrophages). Interestingly, Δ133p53β was shown to directly regulate the mRNA expression of *CD274* that encodes the PD-L1 immune checkpoint marker. Furthermore, prostate cancers with a high level of Δ133p53β mRNA were associated with poor patient outcome [194]. Elevated Δ133p53β was shown previously to be associated with immunosuppressive and chemotherapy-resistant features of glioblastoma [193]. Both chemoresistance and increased metastatic potential can result due to the presence of cancer stem cells (CSC). Recently, Δ133p53β was shown to enhance the cancer cell stemness potential of breast cancer cells and to regulate the expression of *SOX2*, *OCT3/4*, and *NANOG*, key cell pluripotency/reprogramming genes, resulting in increased mammosphere-forming ability and metastatic potential of cancer cells. Furthermore, etoposide treatment of breast cancer cells increased CSC formation and elevated the level of Δ133p53β, as well as *SOX2*, *OCT3/4*, and *NANOG*, which can increase the risk of resistance and cancer recurrence [182]. In addition, Δ133p53 expression was shown to be associated with 5-fluorouracil chemoresistance of cholangiocarcinoma cells [215]. Both Δ133p53 and Δ160p53 isoforms have a truncated DBD that influences the conformation stability of these short isoforms compared to their full-length counterparts and, thus, can alter their function [53]. Most cancer-associated mutations lie within the DBD domain of p53 and can cause a “gain-of-function” (GOF) phenotype characterized by enhanced cell survival, proliferation, invasion, and migration. It has been recently shown that the DBD mutants (e.g., such as p.R175H, p.G245S, p.R248Q, p.R273H, and p.R280K) with oncogenic GOF functions have an overexpressed Δ160p53 isoform. Furthermore, the Δ160p53 isoform was shown to be required for the survival of the DBD mutants after etoposide treatment, and it is responsible for the GOF features (e.g., inhibition of apoptosis, promotion of proliferation, and enhanced adhesion and invasion) observed in DBD mutants. Accordingly, knockdown of the endogenous Δ160p53 isoform was shown to inhibit GOF and to restore apoptosis of DBD mutant cells (i.e., A431 and HT29 cells that express p53 p.R273H) after treatment with thapsigargin, an ER stress-inducing agent [125].

Here, we presented some of the p53 isoforms and their biological activities associated with carcinogenesis and age-related diseases; however, their biological functions in cancer, especially CRC are described later in more detail.

## 8. Biological Activity and Functions of the p73 Isoforms

The p73 protein exerts its functions mainly by acting as a transcription factor affecting the expression of numerous target genes related to tumor suppression, as well as to various non-oncogenic cellular processes [216]. Multiple roles of the p73 protein in crucial processes involved in neurogenesis, sensory pathways, and homeostatic control have been revealed by the global p73-knockout (*Trp73*^−/−^) mice that exhibit severe neurological, pheromonal, reproductive, and inflammatory abnormalities [217]. These mice were created by deletion of exons 5 and 6 of the *Trp73* gene encoding the DBD and were, thus, deficient for the expression of all p73 isoforms. As a consequence, it was impossible to attribute specific biological functions observed to the activity of a particular isoform. Moreover, the initial experiments on *Trp73*^−/−^-mice failed to show increased susceptibility to spontaneous tumorigenesis, most probably due to their short lifespan [2]. Since its discovery and recognition as a member of the pivotal tumor suppressor p53 family, the p73 protein was presumed to be itself involved in tumorigenesis. However, the extremely low frequency of the *TP73* gene mutations in cancer and initial experiments on *Trp73*^−/−^-mice failed to support its potential tumor suppressor role [2]. Later developed heterozygous *Trp73*^+/−^ mutant mice showed increased incidence of diverse spontaneous tumors often manifesting LOH. Moreover, mice heterozygous for both p53 and p73 mutation exhibited an even more aggressive tumor phenotype than the *Trp53*^+/−^ mice bearing only mutation of p53 [218]. These results supported the involvement of p73 in tumor suppression and suggested complex interrelations between the p53 family members. In order to specify the contribution of specific p73 isoforms to the observed phenotypes, several knockout mouse models for particular group of isoforms were consequently developed [219]. The roles of p73 in tumorigenesis and other biological functions were especially complicated to determine due to the existence of TAp73 and ΔNp73, two principal groups of isoforms which generally show distinct functions. TAp73-knockout (*TAp73*^−/−^) mice lacking exons 2 and 3 that encode the TAD showed increased incidence of spontaneous tumors (especially lung adenocarcinoma) and genomic instability (aneuploidy) [220]. At the same time, another group also reported the role of TAp73 in the maintenance of genomic stability as TAp73-knockout MEFs showed increased polyploidy and aneuploidy in the absence of p53 [221].

Maintenance of genomic stability is crucial in cancer prevention, especially upon DNA damage. In parallel with work on knockout models, numerous expression studies on cell models supported the role of p73 isoforms in molecular processes related to tumor suppression such as cell-cycle regulation, DNA repair, apoptosis, and senescence [221,222,223].

The TAp73 isoforms as transcriptional factors are involved in the regulation of the G1/S and G2/M cell-cycle checkpoints, acting as repressors of a plethora of genes (*CDC25B*, *CDC25C*, *CDC2*, *CCNB1*, *CCNB2*, and *TOP2A*) and as inducers of *p21*, *p57*, and *GADD45* [224,225,226]. In addition, the p73 isoforms (TAp73α and ΔNp73α) are important for cell-cycle progression even under normal physiological conditions, as they accumulate during G1/S cell-cycle transition with a peak in the S phase. In cycling cells, both p73 isoforms bind p53RE in the promoters of cell-cycle genes, competing with p53 and impeding its repressing function. Downregulation of p73 with specific siRNAs led to a decrease in the expression of genes involved in G1/S and G2/M progression and a reduction in cell proliferation [227]. TAp73 binds to the promoters and activates the transcription of several genes involved in HR DNA repair, namely, *Rad51*, *BRCA2*, *Mre11*, and *Rad50* [228]. In addition, p73 was found to be induced during DDR caused by bile acids/salts activating genes participating in the BER, such as glycosylases *SMUG1* and *MUTYH* [229].

In the case of excess DNA damage, when the cellular mechanisms are not able to repair the damage, cells are driven to cell death (apoptosis) or permanent cell-cycle exit (senescence). Very early after its discovery, p73 was recognized as an inducer of DNA damage-induced apoptosis, mainly via the transcriptional activation of the components of the extrinsic (CD95, DR4, DR5) and intrinsic (BAX, NOXA, PUMA, BID, p53AIP1, GRAMD4) apoptotic pathways [230,231]. In addition, TAp73 is involved in the induction of apoptosis independently of transcriptional activation through its cleavage by caspase-3 and -8 and subsequent localization to mitochondria [232]. Recently, a transcription-independent role of p73 in the mitochondrial pathway was further supported by the interaction of TAp73 with antiapoptotic factor Bcl-X_L_, which increases apoptosis [233].

The functions of ΔNp73 isoforms are mostly exerted through their dominant-negative effect on TAp73 and wt p53, impeding their activity on numerous target genes. In contrast to *TAp73*^−/−^-mice, ΔNp73-knockout mice expectedly were not prone to tumor formation in agreement with its potentially oncogenic function [234]. ΔNp73 isoforms are found to be upregulated in different cancer types, which often correlates with increased resistance to therapy, aggressiveness, and worse disease outcome. In addition to inactivating p53, ΔNp73 is able to inhibit another important tumor suppressor, Rb, causing its hyperphosphorylation [235]. It was shown that ΔNp73 contributes to cell immortalization by cooperating with oncogene Ras in the process of cell transformation [236]. There are several discoveries that support ΔNp73 contribution to chemoresistance of tumor cells. For example, ΔNp73α induces expression of the *MDR1* gene, also known as *ABCB1*, and its product p-glycoprotein (p-gp) by inhibiting p53 function [237,238]. In addition, ΔNp73 induces the expression of ABCB5 that, as p-gp, is responsible for multidrug resistance [238]. ΔNp73β interacts with the DNA damage sensor protein 53BP1, inhibiting transmission of the DNA damage signal downstream in the DDR cascade, thereby providing another possible mechanism contributing to the tumor’s resistance to therapy [234].

During the last decade, an important role of p73 in the regulation of angiogenesis emerged [239]. While oncogenic ΔNp73 was expectedly found to promote angiogenesis, TAp73 was attributed with both positive and negative regulatory roles [240,241,242,243]. Inhibition of angiogenesis by TAp73 is in agreement with its general tumor suppressor role and is achieved through the MDM2-mediated degradation of the principal angiogenic regulator HIF1-α (hypoxia-inducible factor 1α) [243]. On the other hand, the positive effect of TAp73 on vessel formation could be connected to its increased expression found in several tumors, as well as its role in cell survival, contradicting its typical tumor suppressor role [36,244]. Stabilization of TAp73 in the condition of hypoxia leads to activation of proangiogenic genes, including *Vegf-A* [241].

p73 is extensively implicated in normal and tumor cell metabolism, contributing to adaptation to stress conditions, mainly by regulating the expression of different target genes. TAp73 directly transactivates mitochondrial *Cox4il* (complex IV subunit cytochrome C oxidase subunit 4 isoform 1) involved in mitochondrial metabolism, preventing ROS accumulation and premature aging in mice [245]. Interestingly, TAp73 also contributes to redox homeostasis by facilitating the translation of specific mitochondrial transcripts, affecting the translational machinery itself [246]. High-throughput studies of cancer cell models ectopically expressing TAp73α or TAp73β have confirmed the influence of the TAp73 isoforms on numerous metabolic pathways, both catabolic and anabolic, with seemingly opposite effects [247,248,249,250]. These results confirm that p73 is a multifaceted protein, as some of its metabolic roles, e.g., in glycolysis and anabolic pathways, may be easily interpreted as tumorigenic, contradicting its established tumor suppressor role. On the other hand, Agostini and coauthors suggested that the metabolic changes induced by ectopic expression of the TAp73β isoform may serve as a response to prevent accelerated senescence and aging, and not to promote cellular proliferation [247]. However, further investigations are needed to clarify the meaning of the various metabolic changes caused by the ectopic expression of both TAp73α and TAp73β. TAp73 is also involved in the metabolism of amino acid glutamate, as it regulates the expression of GLS2 (glutaminase 2) that converts glutamine into glutamate [251,252]. Consequently, the loss of TAp73 affects in vivo GABA and glutamate levels in cortical neurons [251], and its presence in medulloblastoma cells leads to a glutamine addiction phenotype, where the lack of glutamine inhibits cell proliferation [252].

The ΔNp73 isoforms have also been implicated in glucose metabolism. Deletion of the ΔNp73 isoforms was shown to inhibit the expression of the glycolysis enzyme hexokinase 2, through upregulation of peptide hormone amylin. This resulted in the inhibition of glycolysis, as well as the induction of ROS and apoptosis, consequently leading to regression of p53-deficient tumors in mice [253]. Accordingly, pramlintide, a synthetic analogue of amylin, causes rapid tumor regression of p53-deficient thymic lymphomas in mice [253]. Furthermore, high-throughput metabolic analysis of *ΔNp73*^−/−^ mice neurons showed slightly reduced glycolysis and reduced amounts of membrane phospholipids, long-chain fatty acids, sphingolipids [254], *N*-acetylaspartylglutamate, and neurotransmitter glycine, suggesting a delay in neuronal differentiation [251]. Interestingly, inhibition of polyamine catabolism decreases the ∆Np73/TAp73 ratio in favor of TAp73 and restores sensitivity of the monocytic leukemic cells THP-1 to doxorubicin [255].

In addition to the numerous functions of p73 more or less closely related to tumorigenesis, during the last decade, many non-oncogenic functions of p73 emerged mainly due to extensive research on the isoform-specific knockout models [219,256]. p73 plays an essential role in neuronal development, with the TAp73 isoform playing a more crucial role in neural development and homeostasis compared to ΔNp73 [256]. TAp73 contributes to the long-term maintenance of neural stem cells via transcriptional regulation of the bHLH Hey2, which prevents their differentiation [257]. On the other hand, TAp73 was found to promote the terminal differentiation of immature neurons through direct induction of p75NTR and miR34a, which in turn signal via RAS–NF-κB–PI3K and Syt1–Stx1A pathways [258,259]. ΔNp73-knockout mice showed only a subtle phenotype in the nervous system [234,260]. Earlier studies showed that the overexpression of ΔNp73α and ΔNp73β rescued mature neurons from apoptosis induced by NGF withdrawal or overexpression of p53 [261]. Furthermore, in vivo, it was found that ΔNp73 plays an essential antiapoptotic role during neuronal death in the developing brain [217,261]. Lastly, recently developed knockout mice with deletion of exon 13 necessary for the α isoforms showed severe hippocampal dysgenesis, reduced synaptic functionality, and impaired learning and memory capabilities, revealing the indispensable role of the C-terminus in normal development of the hippocampus [262].

The TAp73 isoforms are recognized as main transcriptional regulators of multiciliogenesis in airways, oviduct, and brain ependyma. This role explains the airway infections and infertility observed initially in *Trp73*^−/−^ mice, as multiciliated cells are crucial for fluid and germ cell transport across tissue surfaces [263,264,265,266]. In male and female reproduction, TAp73 regulates the gene networks that control cell–cell adhesion programs within the germinal epithelium to enable germ cell maturation.

Different p73 isoforms participate in various cellular processes, many of which are related directly or indirectly to tumorigenesis. Some of these functions are shared between several distinct p73 and p53 isoforms, revealing their potential complementary activities, and some of them are attributed only to a specific isoform. Thus, p53/p73 isoforms form a complex network of different functions and activities which are very often based on protein interactions.

## 9. Crosstalk between p53/p73 Isoforms

Protein interactions are important events in a variety of cellular responses, sometimes causing the loss of protein activity. This is particularly relevant in the context of cancer cells expressing the wt p53 protein (or TAp73), whose functionality is needed for an efficient response to anticancer therapies. Vilgelm et al. showed that p73 and p53 proteins are co-expressed in colon carcinoma tissues from the same patient [37].

**Figure 5:**
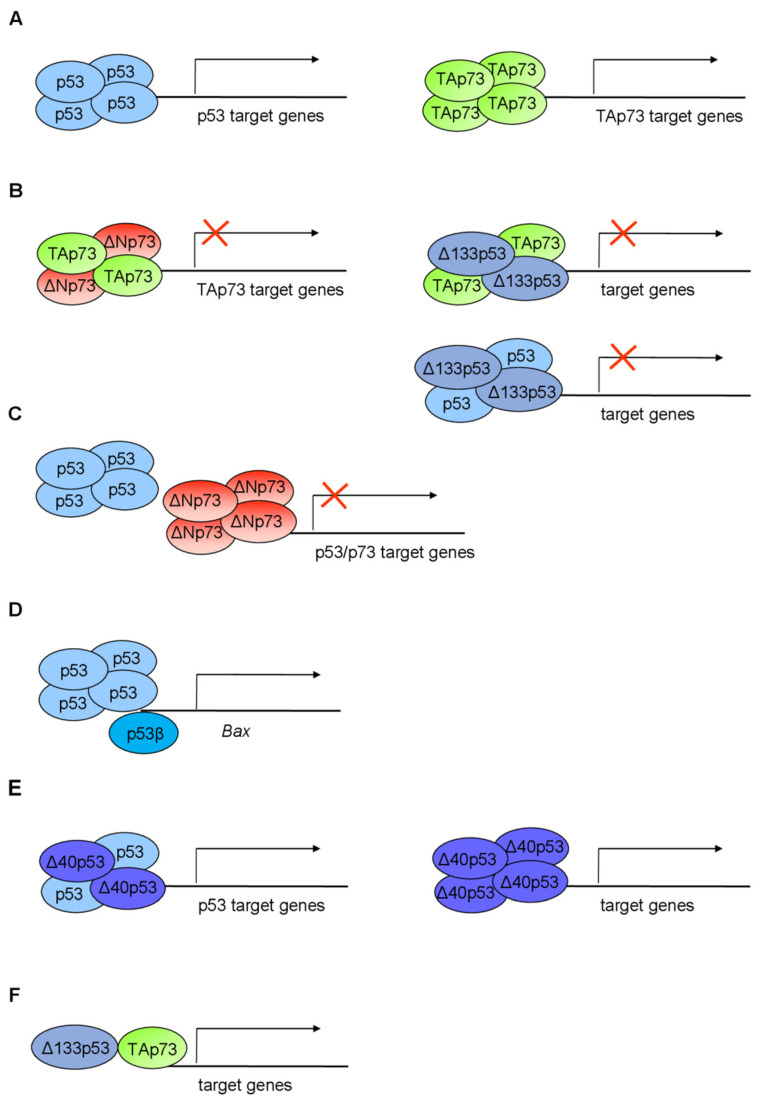
Schematic overview of the p53/p73 isoform interactions mediating transactivation. (**A**) The p53/p73 isoforms form homotetramers to transactivate target genes; (**B**) certain p53/p73 isoforms can form heterotetramers and consequently antagonize p53/TAp73 transactivation ability or (**C**) compete with p53/TAp73 for the same promoter sequences, thus exerting a dominant-negative effect; (**D**) p53β can modulate the promoter activity of target genes (e.g., *BAX*) only in the presence of p53α; (**E**) the p53 isoforms can independently mediate transactivation; (**F**) the p53 isoforms cooperate with the other family members, e.g., p73, to mediate transactivation. Adapted from [14,120,277].

The transactivation function of isoforms of the p53 protein family is executed through the correct formation of a tetramer through the OD of the monomers (Figure 5A). More precisely, it is proposed that the p53 dimer binds to a half-site on the consensus DNA sequence and then is stabilized by binding of the second dimer [267]. In addition, the SAM domain of p73α mediates protein–protein interactions that are essential for tetramerization and, thus, for the formation of the active molecule. The transcriptional activity of the individual tetramer depends on which isoforms it is composed of. Hitherto, we and the others determined physical interactions between certain isoforms of p53/p73 proteins which are involved in carcinogenesis and are an important aspect of cancer research [11,28,31,119,268].

The formation of mixed heterocomplexes between oncogenic and antioncogenic family members correlates with the lack of capacity to transactivate their target genes (Figure 5B). It is one of two mechanisms for the dominant-negative interference with the p53 and TAp73 suppressor functions [4]. Certain mt p53 proteins negatively regulate both wt p53 and TAp73 through a direct physical interaction, which occurs between the DBD of mt p53 proteins and both the DBD and the OD of TAp73. These interactions were confirmed under physiological conditions in mammalian cells [195,269,270], causing the loss of the p53 and TAp73 tumor suppressor functions. Furthermore, mixed protein complexes are formed between endogenous ΔNp73 proteins and either wt p53 or TAp73 in the human tumor cells and clinical tumor tissue, as well as in primary fibroblasts [31,236,271]. The dominant-negative effect of the ΔΝp73 isoforms is performed either through heterocomplex formation with the TAp73 isoforms (Figure 5B) or through competition for promoter binding with both p53 and TAp73 (Figure 5C) [28,31,268]. The latter mechanism was founded by the notion that p73 and p53 do not form heterotetramers due to their structurally diverse ODs [272,273]. In both ways, ΔNp73 efficiently interferes with the p53/TAp73 transactivation function, apoptosis, and growth suppression. When co-expressed in various cell types, ΔNp73 mediates significant stabilization/accumulation of TAp73α/β, while simultaneously inhibiting their transactivation ability [274].

There is an autoregulatory feedback loop among p53, TAp73, and ΔΝp73. Both p53 and TAp73 induce the expression of the ΔΝp73 isoforms via direct binding to the p73-specific RE within P2 [275]. Induced ΔΝp73 in turn negatively regulates the activity of p53 and TAp73 [271,276]. This negative feedback loop is analogous to the p53–MDM2 loop.

We have demonstrated the complex formation between various p53 isoforms (p53β/γ, Δ133p53α/β/γ, Δ40p53α) and TAp73β, while only Δ133p53α/β/γ form a complex with TAp73α. All p53 isoforms counteract the TAp73β transactivation function but with a different efficiency and in a promoter-dependent manner. Interestingly, the TAp73β apoptotic activity was augmented by p53β [119]. It has also been shown that p53β forms a complex with p53α and enhances the p53-mediated apoptosis in H1299 cells, although the canonical OD is partly deleted in p53β isoforms [11]. Later, it was shown that p53β and p53γ coimmunoprecipitate with p53α only in the presence of the p53-responsive promoter, indicating that they modulate p53α transcriptional activity through, at least in part, the formation of DNA-mediated p53α/p53β and p53α/p53γ protein complexes. Hence, although p53β and p53γ lack the OD, they can indirectly interact with p53α (Figure 5D) [91].

Furthermore, the Δ40p53α isoform retains the entire OD and directly interacts with p53α, forming heterotetramers and modifying downstream p53 target genes [14,120]. Consequently, Δ40p53 (also called p47) can act in a dominant-negative manner toward p53α, inhibiting both the p53-mediated transcriptional activity and the p53-mediated growth suppression (Figure 5B) [16,195]. However, in melanoma cells, Δ40p53 via heterocomplex formation activates and directs p53α to promote apoptosis [172]. Moreover, Δ40p53 reduces the MDM2-mediated degradation of p53 [16,278] and influences the p53 cell localization [16]. In addition, Δ40p53 can mediate transactivation independently of the p53α, e.g., Δ40p53 can transactivate *BAX* and *GADD45* in p53-null cells but an oligomeric complex is still not confirmed (Figure 5E) [120].

∆133p53α interacts with p53α, inhibiting p53α-mediated apoptosis, senescence, and G1 cell-cycle arrest but not p53-mediated G2 cell-cycle arrest [19,90]. This is another example of a feedback loop conserved within the p53 gene family, in which p53-induced Δ133p53α modulates the cellular response to the DNA damage. Obviously, the regulation of the p53/p63/p73 internal promoters plays an important role in the biological activities of the p53 family members. However, the dominant-negative effect of ∆133p53 on p53α is not commonly observed and is probably dependent on cell context.

In addition, the direct interaction between family members has been shown in the model organisms. Ou and coauthors have shown that protein–protein interaction between the zebrafish ∆113p53 (a homolog of Δ133p53) and the full-length p53 is essential for the antiapoptotic function of Δ113p53 [173]. Billant and coworkers studied the dominant-negative effect within the p53 family in a model of yeast *Saccharomyces cerevisiae*. All p53 loss-of-function hotspot mutants and transcriptionally inactive isoforms of p53 (Δ133p53α and Δ160p53α) exhibit a dominant-negative potential toward p53α, as well as transcriptionally inactive ΔNp73α and ΔNp73β. The transcriptionally inactive mutants and isoforms have an intact OD and are able to form heterotetramers [279].

Noteworthily, the p53 family members interact with each other to regulate common target genes. In HCT116 colon cancer cells, it was found that p53 and TAp73 isoforms bind simultaneously to *PUMA* and *p21* promoters. Additionally, the transcription activity of the entire network of p53 family proteins contributes to the cellular response to chemotherapeutic drug treatment in colon and esophageal cancer cells [37]. Δ133p53 and p73 synergistically promote the expression of DNA repair genes in a p53-null environment of HCT116 colon cancers cells (Figure 5F) [180]. Flores and coworkers demonstrated that, for the induction of apoptosis, there is an essential teamwork of all family members. Specifically, in the absence of both p63 and p73 (p63/p73^−/−^ double-knockout MEFs), there is no binding of p53 to the promoters of the proapoptotic *BAX*, *NOXA*, and *PERP* genes. However, p73, on the other hand, can induce apoptosis in the cells without functional p53 [280,281].

However, some works support a prion-like p53 aggregation related to the dominant-negative effect of some hotspot mt p53 [282]. The mutations destabilizing the p53 DBD generate coaggregation with wt p53, p63, and p73 [283]. Following the finding that p53 associates into amyloid-like aggregates, an ELISA system based on a polyionic, high-molecular-weight ligand that specifically captures aggregated oligomers and amyloid proteins was developed. Naturally occurring tetramers of the p53 are not bound, but high-molecular-weight aggregates are detected in the ovarian cancer cell lines and patient-derived tumor tissues [284]. Such aggregates could be potential targets for the development of novel therapeutic strategies against tumors.

To conclude, there is a significant crosstalk between family members in the tumors. They can bind specifically to the p53RE and modulate the expression of the p53 target genes. Through binding to the p53RE, some family members can exert dominant-negative effects on the activity of p53, p63, and p73.

## 10. Alterations of the p53 and p73 Isoforms in Cancer

The p53/p73 isoforms have been identified in many different normal and cancer cells and tissues, including, but not restricted to, ovarian, melanoma, bladder, sarcoma, breast, kidney, and colon cancer. Their expression has often been found deregulated in tumors when compared to healthy tissues, which points to their involvement in tumorigenesis [14,28,145,285]. Here, a few examples are illustrated to accentuate their frequent occurrence and importance among different tumor types.

Research on ovarian cancer, for example, has shown that the expression of different p53 isoforms is altered in around 50% of ovarian cancer cell lines and primary ovarian cancers. More so, expression of p53δ, a splice variant caused by a splice site mutation in intron 9, was associated with impaired response to platinum-based chemotherapy and has been identified as a prognostic marker for the recurrence-free and overall survival of ovarian carcinoma patients. p53β was also correlated with adverse clinical features and worse recurrence-free survival in patients exhibiting functionally active p53. Among p73 isoforms, ΔN′p73 was the preferentially expressed ΔN isoform in ovarian cancer cells with functionally active p53 [286]. ΔN′p73 was also found to be overexpressed in ovarian cancer in another study where about one-third of samples exhibited upregulation of TAp73 and to a lesser extent ΔNp73. The correlation analysis pointed toward better overall survival in patients with low expression of ΔN′p73/ΔNp73 [287]. Zaika and colleagues showed that the ΔNp73 is overexpressed in a panel of different tumors but not in normal tissue [28]. Studies on renal cell cancer (RCC) showed that, in wt p53 tumors, the expression of Δ133p53 was downregulated compared to normal adjacent tissue [288]. Unlike in other tumors, p53 is rarely mutated in melanoma, but still fails to protect melanocytes from tumorigenesis. It was hypothesized that the p53 family isoforms might have a role in this phenomenon. Ozretić and colleagues tested this hypothesis by investigating the expression profile of p53/p73 family isoforms in melanoma cell lines and metastatic melanoma samples. The tumor samples showed lower expression of Δ40p53β and ΔNp73 mRNA and ΔNp73β protein, as well as elevated protein levels of Δ133p53α, Δ160p53α and ΔNp73α. Moreover, the higher expression of Δ133p53β and p53α mRNA and the lower expression of p53β mRNA were connected to shorter overall survival [9].

## 11. Alterations of the p53/p73 Isoforms Expression in Colorectal Cancer

The p53 family is expressed as an intricate network of isoforms in both normal and tumor colorectal tissue. Recently conducted genome-wide alternative splicing analysis on the CRC cohort from TCGA database emphasized the importance of alternative splicing events in the p53 signaling pathway in CRC metastasis and recurrence [289]. Several papers have been published supporting this conclusion.

A high expression of the canonical p53, Δ133p53α and β, a moderate expression of Δ133p53γ, and a lower expression of p53β and γ have been reported in normal colon tissue [11]. Colon adenoma tissues express elevated levels of p53β and reduced levels of Δ133p53 isoforms compared to non-adenoma and normal colon tissue. However, the expression of Δ133p53 increases from stage I to II, whereas that of p53β decreases from stage II to III in the colon carcinoma, displaying an interesting dynamic during cancer progression [19]. In agreement with this, the high upregulation of Δ133p53 transcript level in colon cancers was also found in another study [184].

In normal colon, a low gene and protein expression of both TAp73 and ΔNp73 was found compared to the colorectal adenocarcinomas. Interestingly, the prevalence of p73α and β gene expression was shown by nested PCR, compared to p73γ, δ, ϕ, and ε in both normal and tumor tissue [37]. Another study reported primarily the mRNA expression of ΔNp73 in normal and premalignant tissue. However, in CRC, the expression of both isoforms was elevated [290]. Accordingly, strong cytoplasmic ΔNp73 protein staining was detected in normal rectal tissue [291]. Colon cancer tissue showed increased co-expression of TAp73 and ΔNp73 compared with normal epithelial colon tissue [37,290,292], indicating the involvement of both isoforms in colon carcinogenesis. Of note, ΔNp73 and ΔEx2/3p73 expression increased with cancer stage [137,293,294]. ΔNp73 protein staining was increased in rectal cancer tissue compared to normal rectal tissue [291], as well as in γ-irradiation-resistant colon cancer cell line KM12C [295].

ΔNp73β mRNA has also been identified as an exosomal cargo from ΔNp73β ectopically expressing HCT116 colon cancer cells, and it is selectively packed into exosomes from CRC patients when compared to exosomes from healthy controls. The transmission of exosome cargo to epithelial, fibroblast, and endothelial cells and its significant effect on proliferation and drug resistance in the acceptor cells were observed. This finding supports the potential prognostic value of the exosomal ΔNp73 in colon cancer patients [292].

These data paint a complex image of the p53/p73 isoform network in colon healthy tissues, as well as colorectal tumors.

## 12. Involvement of the p53/p73 Isoforms in CRC Development and Progression

**Figure 6:**
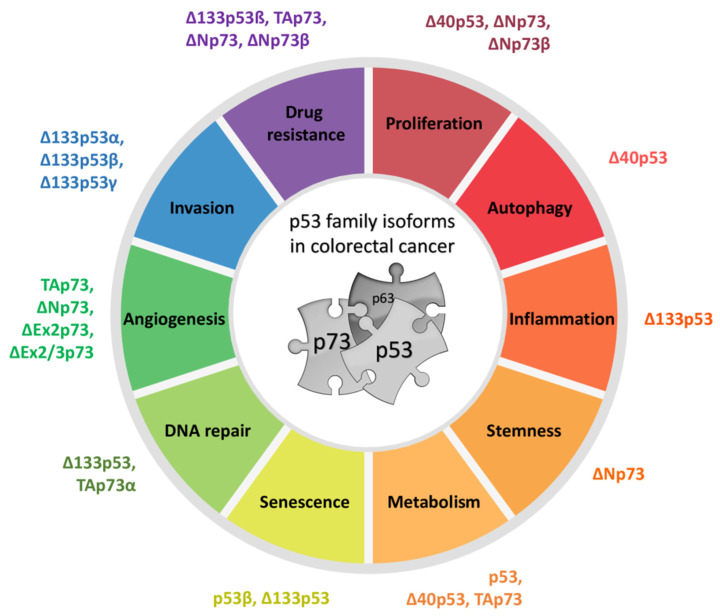
Biological functions of the p53/p73 family isoforms in colorectal cancer. The p53 and p73 isoforms have been implicated in many biological roles in colorectal cancer, namely, drug resistance, proliferation, autophagy, inflammation, stemness, metabolism, DNA repair, angiogenesis, and invasion. Here, the functions are organized in a circular fashion with isoforms associated with a certain biological role listed next to the roles. See Table 1 for details on molecular pathways and references.

Since the p53 family proteins are involved in a plethora of cell signaling pathways that regulate processes such as survival, proliferation, differentiation, apoptosis, and motility of both healthy and cancer cells [4,296,297,298], it is of no surprise that particular isoforms are implicated in CRC as well (Table 1). However, to our knowledge, roles of the p53 isoforms have still not been investigated thoroughly in CRC (Figure 6).

Among the few papers tackling the role of p53 isoforms in colorectal cancer, Katoch and colleagues showed that ∆40p53 downregulates the expression of the transcriptional repressor protein YY1 via upregulating miR-186 which consequently inhibits cell proliferation of HCT116^−/−^ cells [187]. There is also evidence of Δ40p53’s involvement in autophagy in the Δ40p53 ectopically expressing HCT116 cells. More specifically, it was shown that Δ40p53 has 3′–5′ exonuclease capacity, which contributes to its role in inhibiting autophagy [186]. Arsic and colleagues showed that the ∆133p53β isoform specifically binds to a tumor suppressor RhoB (Ras homolog family member B) and negatively regulates its GTPase activity, which in turn protects CRC cells from camptothecin-induced apoptosis [175]. Δ133p53 in complex with TAp73α has been implicated in DNA DSB repair upon γ-irradiation in HCT116 cells. In more detail, experiments showed that these two isoforms accumulate 24 h post irradiation and synergistically cooperate to promote the expression of *RAD51*, *LIG4*, and *RAD52* by binding to their promotors. Furthermore, the knockdown of TAp73 causes accumulation of DNA DSBs, which leads to G2 cell-cycle arrest and promotes cell senescence upon γ-irradiation. Interestingly, mutations in the Δ133p53 promoter region reduce its activation in response to DNA damage, which may be related to tumorigenesis [180]. Diaz and colleagues directly associated the TAp73 and ∆Np73 isoforms with antiapoptotic protein survivin in colon cancer cells. Specifically, experiments on SW480-ADH and HCT116 cell lines showed that 1α,25-dihydroxyvitamin D3 treatment significantly downregulated survivin, which led to a decrease in TAp73 and ∆Np73. On the other hand, the ectopic expression of survivin caused an increase in TAp73 and ∆Np73, as well as ∆Ex2p73 and ∆Ex2/3p73, transcripts. Furthermore, direct correlations between ΔNp73, ΔEx2p73, or TAp73 isoforms and survivin levels were observed in colon cancer tumor samples, pointing to their antiapoptotic role [299]. On the other hand, Dabiri and colleagues implicated the TAp73 isoform as a proapoptotic actor in CRC cells lacking functional p53. Using p53-null HCT116 cells, the authors showed that the transient resistance to inhibitor bortezomib is abolished by the nuclear translocation of TAp73, which causes apoptosis. Similar results were observed in mt p53 HT-29 cells, but not in human fibroblasts expressing wt p53, indicating that TAp73 could be a potential therapeutic target for the treatment of CRC lacking functional p53 [300]. The ectopic expression of ΔNp73 isoforms in HCT116 cells was associated with an increase in proliferation and drug resistance to oxaliplatin [293].

Further experiments confirming the oncogenic role of the ΔNp73 isoforms in CRC showed that ΔNp73 overexpression increased the colonosphere formation of CRC cells DLD1 and significantly reduced cell death in HCT116 and DLD1 caused by a small-molecule prodigiosin, which restores the p53 pathway [301]. Interestingly, when ΔNp73α was overexpressed in wt p53 HCT116, it did neither affected cellular/tumor growth nor the response to anticancer treatment with cisplatin or doxorubicin in vitro and in vivo [302,303]. Similar results were obtained in another study on p53-null HCT116 and H1299 cell lines with stable overexpression of ΔNp73α [304]. Lööf and colleagues showed that the overexpression of ΔNp73β increased cellular viability but did not affect cellular susceptibility to cisplatin in both wt p53 and mt p53 HCT116. Interestingly, cisplatin decreased the expression levels of ΔNp73β in a dose-dependent manner [305]. Furthermore, the exosomal ΔNp73β mRNA from HCT116 cells ectopically expressing ΔNp73β increased proliferation and oxaliplatin chemoresistance in vitro in recipient cells. Moreover, the HCT116 xenografts in mice injected with exosomes from the ΔNp73β-overexpressing cells were significantly bigger compared to controls, showing a higher percentage of proliferative marker Ki67-positive cells and a higher level of the ΔNp73β expression [292].

Experiments have revealed that the p53 isoforms, particularly Δ133p53, are involved in the invasion of CRC. Campbell and colleagues showed that all three Δ133p53 isoforms (α, β, and γ) promote invasion of HCT116 through matrigel via RhoA and ROCKA (Rho-associated protein kinase A). Furthermore, cells transfected with Δ133p53α display a more rounded phenotype [188]. Similarly, ∆133p53 is expressed in higher amounts in the invasive CRC cell lines independently of the p53 mutational status. Additionally, HCT116 cells depleted of all p53 isoforms other than Δ133p53 become more invasive, while those transfected with siRNA specific for p53β isoforms become less invasive in vitro, suggesting that the p53 isoform ratio, including ∆133p53β, defines the invasive activity of the cells. Interestingly, the ectopic expression of ∆133p53β in HCT116 cells initiated epithelial–amoeboid transition, a derivative of epithelial-mesenchymal transition, which produces highly invasive cells [192].

The p73 isoforms may have a role in modifying the expression levels of the angiogenesis-related factors in CRC, which is yet to be understood. Díaz and coauthors found correlations between the expression levels of the proangiogenic VEGF-A (vascular endothelial growth factor A) and ΔNp73 and TAp73 in a cohort of CRC patient samples. Interestingly, the expression of ∆Ex2p73, ∆Ex2/3p73, ∆Np73, or TAp73 correlated with the expression of a putative antiangiogenic isoform VEGF165b. However, the expression of ΔEx2p73 inversely correlated with the antiangiogenic protein PEDF (pigment epithelium-derived factor) [306].

p53 isoforms have been implicated in the cellular metabolism of CRC. For example, glucose starvation promotes IRES in lung and colon cancer cells that is regulated via the SMAR1 (scaffold/matrix attachment region-binding protein 1) protein and elevates the expression of TIGAR (TP53-induced glycolysis regulatory phosphatase) whose product is involved in glycolysis [109]. In addition, higher glycolytic activity was observed in the colon CSCs expressing TAp63 compared to the colon CSCs expressing ∆Np63, which implies that the p63 isoforms regulate their glucose metabolism [309]. Furthermore, TAp73 activates the expression of the pentose phosphate pathway enzyme glucose-6-phosphate dehydrogenase (G6PD), which supports tumor cell proliferation in both p53 wt and ^−/−^ HCT116 [307,308].

Furthermore, the ectopic expression of Δ133p53 in HCT116 cells increases the expression of the NF-κB targets interleukin 6, 8 and antiapoptotic protein Bcl-2, probably through inhibition of p53 [93].

p53β and Δ133p53 show dynamic correlation in the development of colon carcinoma. Increased levels of p53β and reduced levels of Δ133p53 in colon adenoma tissues have been described as a senescence-associated p53 isoform signature that may represent a senescence barrier observed in premalignant tumors. Interestingly, in CRC tissues, this signature is lost and even reversed with cancer stage, which may indicate an escape from the senescence barrier during the progression from adenoma to carcinoma [19].

## 13. Prognostic Relevance of the p53/p73 Isoforms Expression in CRC

The mutation status of an established tumor suppressor protein such as p53 has surprisingly limited relevance in cancer progression diagnostics. One reason might be the overly simplified image of its action, neglecting the existence of the p53 family isoforms which act in concert. Therefore, the question is whether the p53 family isoforms could have a better diagnostic ability for cancer patients than the canonical protein itself. In that context, Campbell and colleagues showed that the CRCs with elevated Δ133p53 mRNA are more aggressive and are associated with a shorter disease-free survival (DFS) [188]. Moreover, high expression of the Δ133p53β mRNA is correlated with higher risk of metastatic recurrence in patients with locally advanced rectal cancer [175]. There is still a large area to explore in the context of potential prognostic value of the remaining p53 isoforms in CRC. On the other hand, more extensive research on the prognostic relevance of the p73 isoforms has already been undertaken. The overexpression of the p73 variants is mostly associated with adverse colon tumor features. More specifically, an association was found between the expression of ∆Np73 or ∆Ex2/3p73 and the CRC tumor stage, as well as between the upregulation of ∆Ex2p73, ∆Ex2/3p73, or TAp73 and the presence of polyps [137,294]. The ∆Np73 overexpression was also associated with vascular invasion of CRC and high levels of the ∆Ex2/3p73 variant with lymph node metastases [294]. Colon cancer patients showed reduced DFS associated with ΔNp73 [310], and the overexpression of ΔNp73 and ΔEx2/3p73 isoforms was associated with advanced stages, as well as shorter OS and DFS [292,293]. ΔNp73 expression was also related to the local recurrence of rectal cancer [293]. However, the same authors revealed a correlation of ∆Np73 expression and long survival time of rectal cancer patients who were subjected to preoperative radiotherapy [311]. Interestingly, higher levels of ΔNp73 in patient-derived exosomes were also noted in sera of advanced-stage CRC patients and were associated with shorter DFS [292]. In conclusion, most papers correlate malignant characteristics of colon cancer with the elevated expression of ΔNp73 isoforms.

Findings have shown that p53 autoantibodies are produced early during tumor development in CRC patients, making the p53 autoantibodies promising markers for early detection [312,313]. Research on seropositivity extended from the canonical p53 protein to p53 family isoforms has been done in CRC patients. More specifically, sera were tested for total p73, ΔNp73α, and ΔNp73β autoantibodies, and results revealed higher ∆Np73α and ∆Np73β seroreactivity of CRC samples when compared to healthy individuals. Importantly, autoantibodies to both isoforms showed diagnostic ability separately and even better in combination in discriminating between CRC and healthy individuals. Interestingly, the ∆Np73 isoform autoantibodies showed even higher diagnostic ability than the canonical protein TAp73α [290].

These data point to the importance of investigating isoforms hand in hand with the canonical proteins themselves in cancer research.

## 14. Targeting the p53/p73 Family Isoforms in CRC

Since there is ongoing evidence that p53 family isoforms act as epigenetic p53 inhibitors in cancer, the potential of targeting them in colon cancer has been investigated, with the goal of reactivating the p53 pathway and getting a better response to therapy. Zhang and colleagues published an interesting article where they, after a functional cell-based assay screening of almost 2000 small molecules, identified NSC59984 as that which specifically restores the p53 pathway in mt p53 CRA cells SW480 and DLD-1 via activation of TAp73. Importantly, a favorable therapeutic index between normal and cancer cells was presented. The authors showed that NSC59984 not only induces TAp73-dependent cell death in cancer cells in vitro but also synergizes with the DNA-damaging agent CPT11 in inducing cell death of mt p53-expressing cells [314]. Furthermore, β-cryptoxanthin, a carotenoid contained primarily in citrus fruits, has shown an effect on p53 family signaling. More precisely, β-cryptoxanthin treatment in vitro upregulated TAp73 and downregulated ΔNp73, ΔEx2/3p73, and ΔEx2p73 mRNA levels in most of the colon cancer cells investigated. An intervention study in humans showed that intake of β-cryptoxanthin-enriched juice also affected the expression of p73 isoforms in leukocytes, where significant downregulation of ΔNp73 has been found. Furthermore, β-cryptoxanthin decreased the proliferation of colon cancer cells and HCT116 xenografts and enhanced the effect of oxaliplatin in inducing apoptosis and a reduction in tumor volume [315]. Nutlin-3, an already well-known small-molecule inhibitor, which activates p53 by disrupting the p53–MDM2 association, has also been tested in the p53-null CRC background. When p53 is absent from HCT116 cells, nutlin-3 inhibits MDM2 binding to TAp73α, which leads to the upregulation of TAp73 protein levels, prolongation of the p73 half-life, and an increase in its transcriptional activity. Consequently, the transcription of p73 target genes *NOXA*, *PUMA*, and *p21* leads to an increase in apoptosis [316]. Interesting research has also been done on colorectal cancer stem cells (CRCSC), a rare self-renewing cell subpopulation which adds a further layer of complexity to cancer treatment because they contribute to tumor maintenance and chemoresistance. Prabhu and colleagues showed that a small-molecule prodigiosin, which restores the p53 pathway in cancer cells, is also effective on 5-FU chemoresistant CRCSCs in vitro and in vivo. More specifically, prodigiosin increases the level of TAp73 and decreases ΔNp73 in a c-*Jun*-dependent manner. This research highlighted the involvement of the p73 pathway in the maintenance of stem potential of CRCSC, as well as the potential of p53 pathway-activating therapeutics to target colorectal cancer [301].

Since p53 isoforms are differentially expressed in CRA and CRC and correlated to patient prognosis, their targeting to restore the p53 signaling pathway alone or in combination with already established anticancer agents presents an attractive strategy for CRC therapy.

## 15. Conclusions

The p53 family members play a significant role in carcinogenesis and treatment response. In addition to the fact that p53 is often mutated in tumors, it has become evident that different expression patterns of its isoforms are relevant in carcinogenesis. Furthermore, in tumors with mutated or otherwise inactivated p53, substituting its tumor suppressor function by TAp73 is a possible therapeutic approach. However, the existence of p53-like and p53-inhibitory isoforms, as well as a close functional crosstalk among all family members, gives the p53 family isoforms both tumor suppressor and oncogenic roles. To completely understand and decipher the role of the p53 and p73 isoforms in cancer, it is important to explore the mechanism supporting the dysregulation of isoform balance, as well as perform clinicopathologic studies which correlate the co-expression of p53 family isoforms with p53 mutation status and clinical outcome. Once the complex regulation of the p53 family isoforms is fully understood, they can be used as biomarkers or as therapeutic options.

## Figures and Tables

**Table 1:** Involvement of the p53/p73 isoforms in colorectal cancer development and progression. The p53/p73 family of isoforms is associated with a plethora of cancer-related cellular functions such as apoptosis and sensitivity to drugs, DNA repair, autophagy, proliferation, inflammation, invasion, angiogenesis, metabolism, and senescence.

Cellular Function	Isoform	Model	Signalling	Effect	Reference
Apoptosis and sensitivity to antitumor drugs	∆40p53	HCT116^−/−^	↑ miR-186 → ↓ YY1	↓ proliferation	[187]
	∆133p53ß	HCT116, SW480, LoVo, SW620 and Colo205	↓ RhoB	↑ resistance to camptothecin-induced apoptosis	[175]
	TAp73, ∆Np73, ∆Ex2p73, ∆Ex2/3p73	SW480-ADH and HCT116	↑ survivin → ↑ p73 isoforms	-	[299]
	TAp73	HCT116 p53^−/−^ and p53-mt HT-29	Casp3 and PARP cleavage	↑ bortezomib-induced apoptosis	[300]
	ΔNp73	HCT116	-	↑ proliferation and resistance to oxaliplatin-induced apoptosis	[293]
	ΔNp73	DLD1 and HCT116	-	↑ colonosphere formation and resistance to prodigiosin	[301]
	ΔNp73α	HCT116 and HCT116 xenograft	-	= cellular and tumor growth = sensitivity to cDDP or DX in vitro and in vivo	[302,303]
	ΔNp73α	HCT116 p53^−/−^	-	= cellular growth = sensitivity to cDDP, DX and UV light	[304]
	ΔNp73β	HCT116 and HCT116 p53^−/−^	-	↑ cellular viability = sensitivity to cDDP	[305]
	Exosomal ΔNp73β	HCT116 and HCT116 xenografts	↑ ΔNp73β mRNA in recipient cells	↑ proliferation and resistance to oxaliplatin in vitro ↑ proliferation and tumor size	[292]
Autophagy	Δ40p53	HCT116	↓ PKR/ Eif2 and DRAM	↓ starvation and methyl methane sulfonate-induced autophagy	[186]
DNA repair	Δ133p53 and TAp73α	HCT116	↑ RAD51, LIG4 and RAD52	DNA double-strand break repair upon γ-irradiation	[180]
Inflammation	Δ133p53	HCT116	↑ IL-6, IL-8, Bcl-2	-	[93]
Invasion	∆133p53 and Δ133p53β	HCT116, LoVo, SW480, SW620, Colo205	↓ E-cadherin and β1-integrin	↑ invasion ↑ rounded phenotype ↓ adhesion ↑ epithelial–amoeboid transition	[192]
	Δ133p53α, Δ133p53β and Δ133p53γ	HCT116	↑ RhoA and ROCKA	↑ invasion ↑ rounded phenotype	[188]
Angiogenesis	TAp73, ΔNp73	CRC patient samples	Correlations with VEGF and VEGF165b	-	[306]
	∆Ex2/3p73		Correlation with VEGF165b		
	∆Ex2p73		Correlations with VEGF and VEGF165b Inverse correlation with PEDF		
Metabolism	p53 and Δ40p53	HCT116 and A549	Glucose starvation → ↑SMAR1 ↑ IRES ↑ TIGAR	-	[109]
	TAp73	HCT116 and HCT116 p53^−/−^	↑ G6PD	↑ proliferation	[307,308]
	TAp63	Patient-derived CCSCs	-	↑ glycolytic activity	[309]
Senescence	↑ p53β and ↓ Δ133p53	CRA patient samples	-	↑ senescence	[19]
	↓ p53β and ↑ Δ133p53	CRC patient samples		Escape from senescence barrier	

↓, decreased activity, expression, or effect; ↑, increased activity, expression, or effect; →, leads to; =, unchanged; Casp3, caspase 3; CCSCs, colon cancer stem cells; cDDP, *cis*-diammine-dichloro-platinum; CRA, colorectal adenoma; CRC, colorectal carcinoma; DRAM, damage-regulated autophagy modulator 1; DX, doxorubicin; Eif2, eukaryotic initiation factor 2; G6PD, glucose-6-phosphate dehydrogenase; LIG4, DNA ligase 4; IL-6, interleukin 6; IL-8, interleukin 8; IRES, internal ribosome entry site; PEDF, pigment epithelium-derived factor; PKR, protein kinase R; RhoB, Ras homolog family member B; TIGAR, TP53-induced glycolysis regulatory phosphatase; VEGF, vascular endothelial growth factor.
